# Mechanistic diversity within RLC-dependent myosin ATPase inhibitors differentiates EDG-7500, a diastolic-selective cardiac sarcomere modulator

**DOI:** 10.1172/jci.insight.203086

**Published:** 2026-09-22

**Authors:** Craig A. Emter, Marcus Henze, Mike DuVall, Sarah Lehman, Lindsey Lee, Ben Barthel, Natalie A. Hawryluk, Molly Madden, Yangsong Wu, Amy Perry, Martin Beyer, Eric Wei, Cassady Rupert, Steve Roof, Angela Peter, Breanne L. Newell-Stamper, Ying Qian, Emily DiNatale, Sara Cantrell, Jessica Tolley, Stephen Schlachter, Jolanda van der Velden, Michelle Michels, Christine Seidman, Weikang Ma, Leslie Leinwand, Stuart Campbell, Julien Ochala, David Bluemke, Darla Tharp, Jonathan Seidman, Carlos L. del Rio, Marc Semigran, Marc Evanchik, Kevin Koch, Alan Russell

**Affiliations:** 1 Edgewise Therapeutics, Boulder, Colorado, USA.; 2 Department of Molecular, Cellular, and Developmental Biology, University of Colorado, Boulder, Colorado, USA.; 3 Department of Genetics, Harvard Medical School, Boston, Massachusetts, USA.; 4 Department of Cardiovascular Surgery, University Heart & Vascular Center Hamburg-Eppendorf and German Center for Cardiovascular Research (DZHK), Partner Site Hamburg/Kiel/Lübeck, Hamburg, Germany.; 5 Propria LLC, Branford, Connecticut, USA.; 6 Qtest Labs, Columbus, Ohio, USA.; 7 Department of Physiology, Amsterdam UMC, Vrije Universiteit Amsterdam, Amsterdam, Netherlands.; 8 Erasmus Medical Center, Cardiovascular Institute, Thoraxcenter, Rotterdam, Netherlands.; 9 Department of Biology, Center for Synchrotron Radiation Research and Instrumentation and Pritzker Institute of Biomedical Science and Engineering, Illinois Institute of Technology, Chicago, Illinois, USA.; 10 Departments of Biomedical Engineering and Cellular and Molecular Physiology, Yale University, New Haven, Connecticut, USA.; 11 Biomedical Institute, University of Copenhagen, Copenhagen, Denmark.; 12 Department of Radiology, University of Wisconsin-Madison, Madison, Wisconsin, USA.; 13 Department of Biomedical Sciences and NextGen Precision Health, University of Missouri- Columbia, Columbia, Missouri, USA.

**Keywords:** Cardiology, Muscle biology, Heart failure, Pharmacology, Therapeutics

## Abstract

Small molecules that modulate myofibril ATPase activity via the myosin regulatory light chain (RLC) display a broad spectrum of activity in their ability to enhance relaxation and slow contraction. EDG-7500 exhibits features consistent with a ‘diastolic-selective’ cardiac sarcomere modulator (d-CSM), and its ability to treat HCM was explored in engineered human tissue (EHT), human HCM cardiac strips, and an R403Q mutation swine model. In fibers, EDG-7500 preferentially inhibited myofibril ATPase activity and force at diastolic calcium levels, retained length-dependent force activation, accelerated relaxation, and exhibited a shallow, self-limiting inhibitory-exposure response to LV fractional shortening. Compared to CMIs, EDG-7500 moved myosin heads towards the thin filament and accelerated relaxation without decreasing force in mutated EHTs (R403Q). In human HCM cardiac strips, EDG-7500 did not alter myosin SRX state, but decreased Ca2^+^-sensitivity of force independent of mutation. In R403Q swine, chronic EDG-7500 normalized LV filling pressure and prevented pathological cardiac remodeling while preserving normal systolic function and cardiac reserve. EDG-7500 differentiates itself from CMIs by uniquely targeting both phases of the cardiac cycle, improving ventricular relaxation while preserving systolic function. This suggests optimal diastolic efficacy can be reached without balancing systolic impairment.

## Abstract 2

The cardiac sarcomere modulator EDG-7500 demonstrates diastolic efficacy without systolic impairment differentiating it amongst other RLC-modulators and traditional cardiac myosin inhibitors, offering a novel therapeutic approach for treating both obstructive and nonobstructive HCM and potentially optimizing functional benefit.

## Graphical abstract

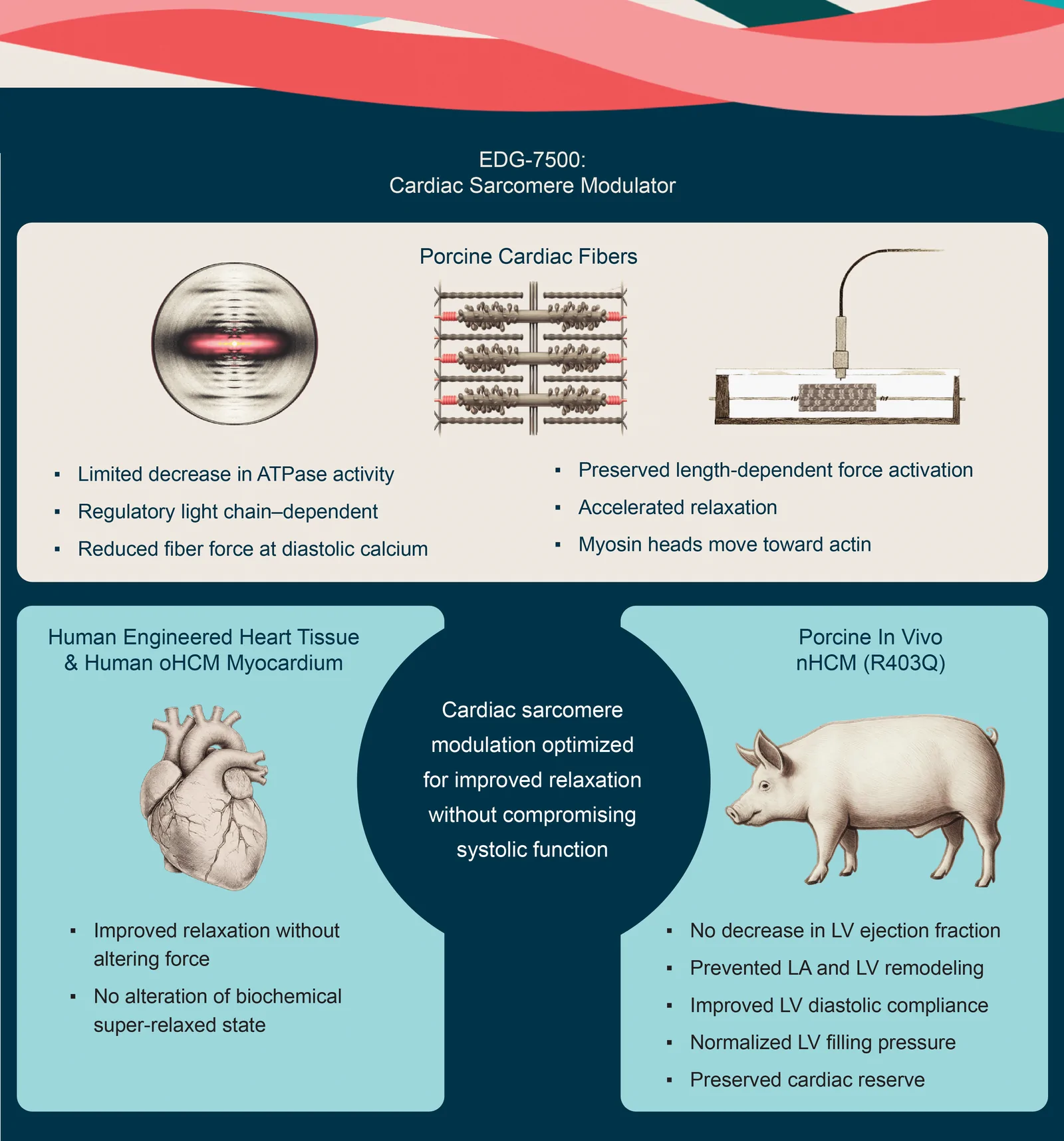

## Introduction

Hypertrophic cardiomyopathy (HCM) is characterized by cardiac hypertrophy in the absence of another systemic, metabolic, or cardiac disease capable of producing a similar level of remodeling in a patient for which a sarcomere or sarcomere-related mutation is identified that could cause such disease or genetic etiology cannot be determined (1). HCM occurs with left ventricular (LV) outflow tract (LVOT) obstruction (oHCM) or is nonobstructive (nHCM) in the presence of concentric cardiac remodeling, with a pathophysiology characterized by hypercontractility and diastolic dysfunction (1, 2). Recently, cardiac myosin inhibitors (CMIs), including mavacamten and aficamten, that target disease pathophysiology have challenged traditional therapies such as β-blockers, calcium channel blockers, and myectomy to treat oHCM. Both CMI compounds decrease LV hypercontractility and LVOT gradient, with long-term data suggestive of positive disease remodeling (3–8). While both therapies have shown clear ability to improve symptoms and function in oHCM, both mavacamten and aficamten decrease inotropy and require careful titration to minimize the risk of excessive LV ejection fraction (EF%) reduction. Furthermore, the recent ODYSSEY-HCM trial reported mavacamten failed to meet both of its primary end points in patients with nHCM (9), suggesting the clinical efficacy of CMIs may be potentially limited to oHCM patients.

We identified, to the best of our knowledge, 2 novel regulatory light chain–dependent (RLC-dependent) cardiac sarcomere inhibitors with remarkably diverse cardiac properties, highlighted by key differences in their ability to enhance diastolic versus inhibit systolic function. The RLC is a thick filament protein that binds to the lever arm of myosin heavy chain, influencing actin-myosin interactions when phosphorylated by increasing force production and Ca^2+^ sensitivity of contraction (10). Over the course of development, we discovered an enantiomeric pair of small molecules, EDG-7499 and EDG-7500, that displayed extremes of this phenotypic diversity. The profile for EDG-7500 demonstrated inhibition of cardiac myofibril ATPase activity and fiber force at diastolic compared with systolic Ca^2+^ levels while retaining length-dependent force activation. EDG-7500 also preferentially accelerated cardiac fiber relaxation versus slowing contraction. In vivo, rat echocardiography demonstrated that EDG-7500 exhibited a shallow, self-limiting inhibitory-exposure response to LV fractional shortening (FS%) accompanied by normal cardiac reserve. In contrast, its enantiomer EDG-7499 displayed properties consistent with CMIs including greater inhibition of myosin ATPase activity across all Ca^2+^ levels, decreased length-dependent activation, preferential inhibition of cardiac contraction versus enhancement of relaxation, and a steep inhibitory-exposure response to LV FS% with blunted cardiac reserve. Recent in vitro reports have described a small molecule similar to EDG-7499 that modulates myosin ATPase indirectly via the myosin RLC (11). Termed an RLC-dependent CMI (RLC-1) (11), this compound also relies on the RLC to inhibit myosin ATPase activity and displays both shared features and distinct differences from traditional CMIs. Given the distinctly divergent properties of these compounds, we termed EDG-7500 a diastolic-selective cardiac sarcomere modulator (d-CSM) and EDG-7499 a systolic-selective CSM (s-CSM).

The entirety of data comparing these enantiomeric pairs provide the rationale to specifically explore EDG-7500 therapeutically in experimental settings of HCM. We hypothesized that EDG-7500 would improve diastolic impairment without diminishing systolic function. Accordingly, the purpose of this study was to: (a) examine the effects of acute EDG-7500 exposure in engineered human tissue (EHT) and human HCM cardiac strips and (b) assess chronic treatment in a preclinical swine model of nHCM. These studies focused primarily on the R403Q mutation in the myosin heavy chain 7 (*MYH7*) gene. In a prevention study, we utilized a Yucatan mini-pig model with a heterozygous *MYH7* R403Q mutation (12–14) that exhibits concentric hypertrophic remodeling of the heart, fibrosis, diastolic dysfunction, and hypercontractility (15). In total, our results point to what we believe to be a new class of small molecule with a biochemical, functional, and therapeutic profile contrasting existing CMIs that could prove beneficial for patients with HCM.

## Results

### Biochemical effects of EDG-7500 and EDG-7499 on myosin ATPase activity.

**Figure 1:**
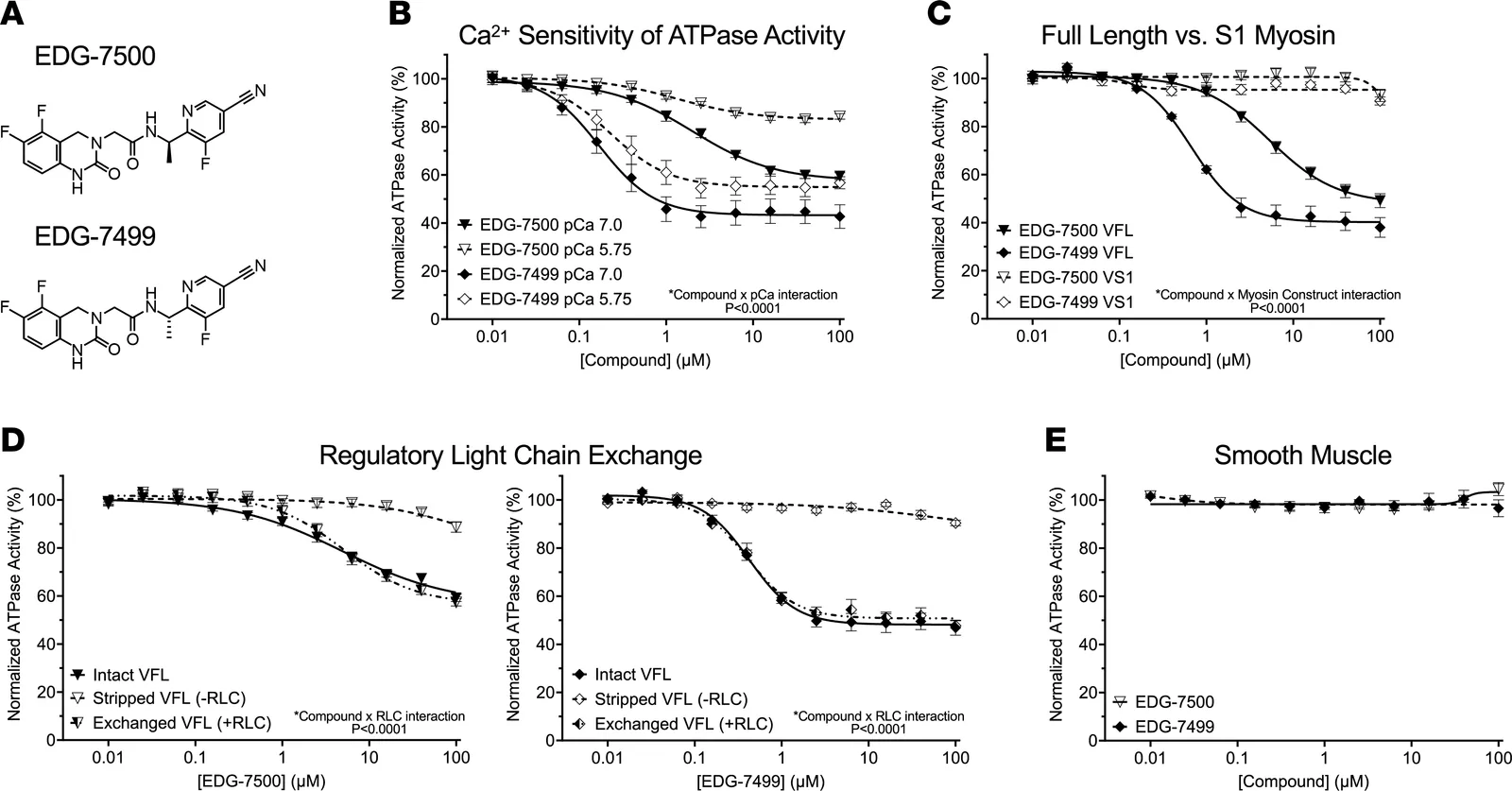
Biochemical assessment of EDG-7500 and EDG-7499 demonstrates preferential inhibition of myofibril ATPase activity at diastolic calcium concentrations and mechanistic action through indirect modulation of enzymatic activity that requires the regulatory light chain (RLC). (**A**) Chemical structure of EDG-7500 and EDG-7499. (**B**) EDG-7500, but not EDG-7499, inhibits porcine left ventricle myofibril DMSO normalized ATPase activity preferentially at diastolic Ca^2+^ (pCa 7.0) compared with systolic Ca^2+^ (pCa 5.75) concentrations (*n* = 6 biological replicates). (**C**) EDG-7500 and EDG-7499 preferentially inhibit DMSO normalized ATPase activity of porcine ventricle full length (VFL) myosin but do not inhibit porcine left ventricle myosin subfragment-1 (VS1) (*n* = 6 biological replicates). (**D**) EDG-7500 and EDG-7499 inhibition of DMSO normalized ATPase activity in porcine VFL myosin requires the regulatory light chain (RLC) (*n* = 6 biological replicates). (**E**) EDG-7500 and EDG-7499 have no inhibitory effects on DMSO normalized ATPase activity in chicken gizzard smooth muscle myofibrils (*n* = 4 biological replicates). *P < 0.05, repeated-measures ANOVA. Experiments **C**–**E** were completed in the absence of Ca^2+^ as purified full-length myosin, proteolytically digested myosin subfragment 1, and unregulated actin do not require calcium activation.

Drug discovery and medicinal chemistry efforts identified what we believe to be 2 novel inhibitors with ventricular myofibril ATPase activity. EDG-7500 and EDG-7499 were identified based on their potency, partial inhibitory profile, and drug-like properties (Figure 1A). The inhibitory effects of each compound were tested in porcine ventricle myofibrils at diastolic (pCa 7.0) and systolic (pCa 5.75) calcium concentrations. Both compounds exhibited a dose-dependent partial inhibition of myofibrillar ATPase activity. Figure 1B shows a significant interaction (compound X pCa), indicating ATPase inhibition for each compound is dependent on calcium level. EDG-7500 showed a pronounced loss of inhibition in the presence of systolic compared with diastolic calcium levels, with a maximum ATPase inhibition of 42% and EC_25_ = 3.0 μM at diastolic calcium levels compared with 18% inhibition and EC_25_ > 100 μM at systolic calcium levels. Comparatively, EDG-7499 had a smaller calcium-dependent shift with 57% inhibition and EC_25_ = 0.18 μM at diastolic calcium compared with 45% inhibition and EC_25_ = 0.39 μM at systolic calcium levels. We considered the decreased effect of EDG-7500 on myosin ATPase inhibition at systolic compared with diastolic calcium levels a more favorable profile by which diastolic effect is achieved without having to compromise systolic enzymatic function. This in contrast to the CMI perspective whereby nearly complete inhibition of myosin ATPase activity at any calcium level maximizes efficacy, implying that the increased and calcium-independent potency of EDG-7499 would appear to be more efficacious. These data highlight significant differences in calcium sensitivity regarding each compound’s ability to inhibit ATPase activity, potentially affecting their ability to balance diastolic efficacy with systolic inhibition in a manner that contrasts current CMIs.

To assess myosin enzymatic activity, compounds were tested in an actin-activated ATPase assay comparing double-headed ventricle full-length (VFL) myosin bound by the essential light chain (ELC) and the RLC to a proteolytically digested single-headed, myosin motor subfragment (VS1). A significant interaction (compound X myosin construct) was observed, showing that ATPase inhibition for each compound is dependent on the myosin construct. Partial inhibitory effects for both EDG-7500 and EDG-7499 were seen in only the myosin full-length system (Figure 1C), with EDG-7499 achieving a greater potency similar to that observed in myofibrils (Figure 1B). Given that these compounds were inactive against the myosin motor domain fragment, these data suggest inhibition is likely exerted on myosin’s ATPase activity indirectly.

Based on the partial inhibition profile and lack of compound effect on myosin motor domain ATPase activity, we hypothesized the RLC may be a necessary structural component for each compound’s mechanism of action. RLC exchange experiments were performed where the RLC was selectively removed from full-length myosin and subsequently replaced with recombinant human RLC protein (encoded by human ventricle *MYL2*; representative gel of the protein exchange is presented in Supplemental Figure 1; supplemental material available online with this article; https://doi.org/10.1172/jci.insight.203086DS1). A significant interaction effect (compound X RLC) demonstrated that inhibition of ATPase by each compound was dependent on the presence of the RLC (Figure 1D). Inhibition of ATPase activity was lost for both EDG-7500 and EDG-7499 when the RLC was absent from the full-length myosin condition. When recombinant RLC was replaced, inhibition and potency were restored to native full-length control values. EDG-7499 and EDG-7500 were inactive against chicken gizzard myofibrils treated with myosin light chain kinase (MLCK), indicating that these compounds do not inhibit smooth muscle ATPase activity (Figure 1E). In total, these data indicate that EDG-7500 and EDG-7499 are allosteric modulators of myosin ATPase activity dependent upon a molecular context, which requires the RLC residing within a fully intact myosin structure.

### Myocardial fiber characteristics of EDG-7500 and EDG-7499.

**Figure 2:**
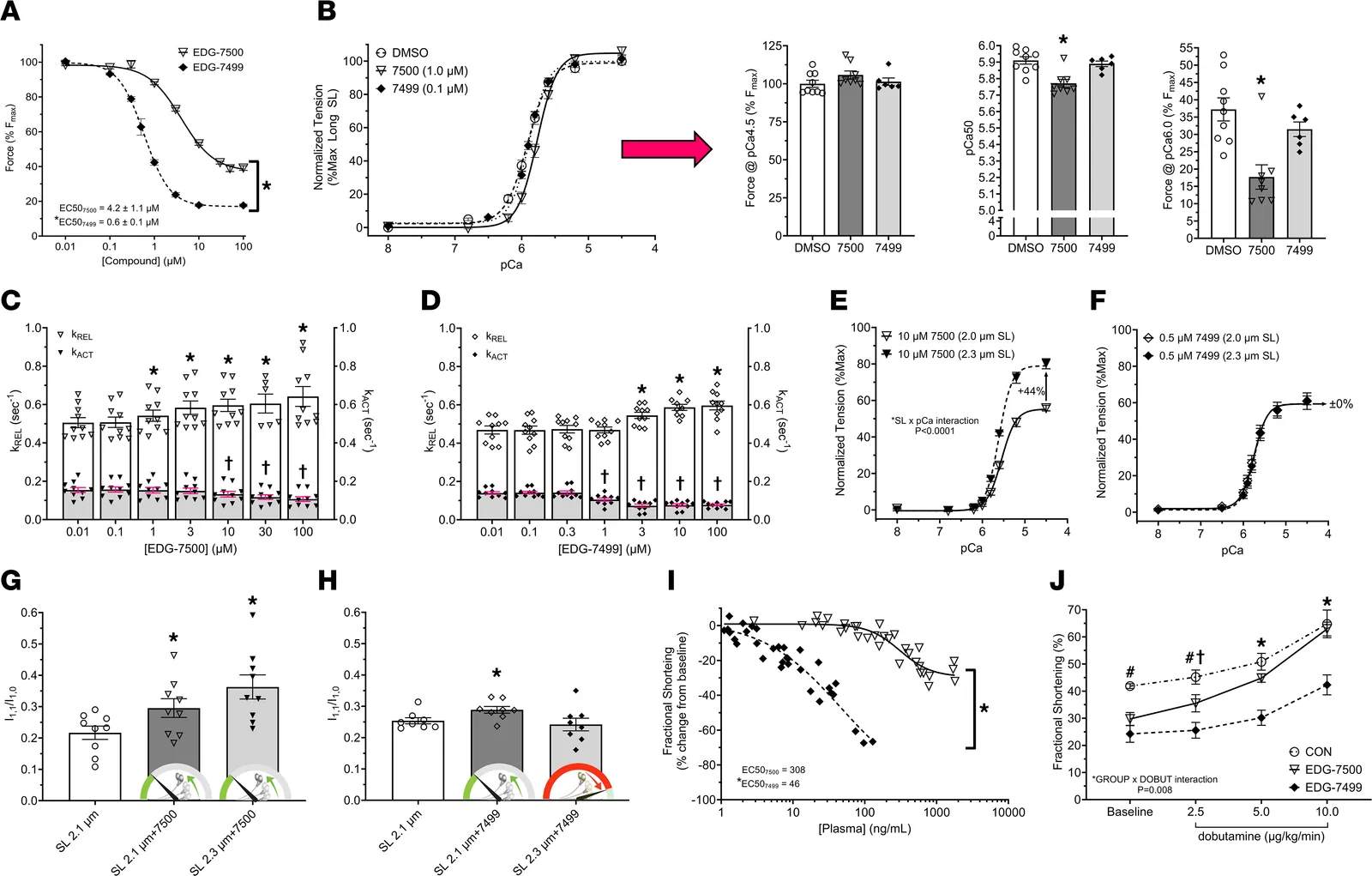
Functional comparisons between EDG-7500 and EDG-7499 highlight a phenotype preferentially promoting relaxation and preserving cardiac reserve without impacting systolic function in EDG-7500 treated tissue and animals. (**A**) EDG-7500 decreases force to a lesser extent in porcine LV skinned fibers (*n* = 5–26). (**B**) Calcium dependence of tension was significantly reduced in EDG-7500–treated fibers with inhibition observed at low levels but not maximally activated Ca^2+^ in porcine LV skinned fibers. (**C**) EDG-7500 activates relaxation kinetics at lower compound dose levels (1 μM; *P < 0.05 versus 0.01 μM k_REL_ dose) prior to decreasing activation (10 μM; ^†^*P* < 0.05 versus 0.01 μM k_ACT_ dose) in porcine LV myofibrils. (**D**) EDG-7499 decreased activation kinetics (1 μM; ^†^*P* < 0.05 versus 0.01 μM k_ACT_ dose) prior to increasing relaxation (3 μM; *P < 0.05 versus 0.01 μM k_REL_ dose) in porcine LV myofibrils. (**E** and **F**) Cardiac myosin length-dependent activation (LDA) indicated porcine LV myofibrils treated with EDG-7500 recovered 44% of an initial force inhibition of ~40% (**E**, *n* = 8). EDG-7499 did not alter LDA (**F,** *n* = 6). (**G** and **H**) EDG-7500 and EDG-7499 significantly increased I_1,1_/I_1,0_ at SL 2.1 (illustrative insets describe myosin head movement) in porcine LV myocardial strips. EDG-7500 preserved a significant increase in I_1,1_/I_1,0_ at SL 2.3 (**G**). Myosin head recruitment was decreased in response to stretch after EDG-7499 treatment (**H**). (**I**) The PK/PD relationship for FS% was significantly shifted to the right in EDG-7500–treated Sprague Dawley rats. (**J**) EDG-7500 preserves normal cardiac reserve in response to β-adrenergic agonism compared with Sprague Dawley rats treated with EDG-7499 (*P < 0.05 CON and EDG-7499 versus EDG-7500; ^#^*P* < 0.05 CON versus EDG-7499 and EDG-7500; ^†^*P* < 0.05 EDG-7500 versus EDG-7499). *P < 0.05, regression analysis for **A**, **B**, and **I**; repeated-measures ANOVA for **C**–**H** and **J**; 1-way ANOVA used for specific pCa assessments presented in **B**.

EDG-7500 and EDG-7499 were tested for force generation across a range of concentrations (0.01–100 μM) in loaded, permeabilized muscle fibers isolated from porcine left-ventricle (Figure 2A). EDG-7500 showed a limited maximal inhibition compared with EDG-7499 (*P* < 0.001; –64.0% ± 4.6% versus –83.6% ± 4.6%) with a significantly lower potency (measured as EC_50_). Based on these curves, concentrations were chosen that were estimated to inhibit force by ~10% (-7% for EDG-7499 at 0.1 μM and –12% for EDG-7500 at 1.0 μM) to test tension development over a range of calcium concentrations (Figure 2B) at comparable levels of peak developed tension. Compared with DMSO at sarcomere length 2.3 μm (SL 2.3), neither compound showed a significant reduction of maximal force at pCa 4.5. EDG-7500 displayed a rightward shift of the force-pCa curve (measured as pCa50) that was observed in parallel with a reduction in force at submaximal Ca^2+^ levels (pCa 6.25–5.75 versus no difference in tension at pCa 5.5–4.5) compared with DMSO and EDG-7499–treated fibers.

Rates of relaxation (k_REL_) and activation (k_ACT_) were measured in porcine left-ventricular bundles of myofibrils across a range of concentrations. EDG-7500 increased the rate of relaxation at concentrations starting at 1 μM, which occurred prior to significant decreases in activation that started at 10 μM (Figure 2C). In contrast, Figure 2D shows EDG-7499 caused significant decreases in activation (1 μM) prior to changes relaxation rate (3 μM). These data demonstrate increases in the rate of relaxation for EDG-7500 precedes the slowing of contraction by a 10-fold concentration window in contrast to EDG-7499, in which the slowing of contraction precedes the speeding of relaxation by a 3-fold concentration.

EDG-7500 and EDG-7499 were tested for their respective sensitivity to length-dependent activation. Compound concentrations were chosen to reduce force to similar levels (–44.2% ± 4.4% at 10 μM for EDG-7500 and –39.1% ± 11.1% at 0.5 μM for EDG-7499) at SL 2.0. Lengthening the muscle to SL 2.3 resulted in a 44% increase in force for EDG-7500 (Figure 2E), while no significant increase in force was observed for EDG-7499 (Figure 2F).

Length-dependent activation was also tested in permeabilized porcine muscle fibers by small-angle x-ray diffraction to determine the recruitment of myosin head density in response to 10 μM EDG-7500 and EDG-7499. Both EDG-7500 and EDG-7499 significantly increased I_1,1_/I_1,0_ (an indicator of myosin head proximity to the thin filament) at SL 2.1 (Figure 2, G and H), indicating increased recruitment of myosin heads toward the thin filament independent of SL 1. When muscle fibers were lengthened to SL 2.3, EDG-7500 continued to show a significant increase in I_1,1_/I_1,0_ compared with DMSO at SL 2.1 (Figure 2G). In contrast, compared with SL 2.1 myosin head recruitment was significantly decreased when muscle fibers were lengthened to SL 2.3 in the presence of EDG-7499 (Figure 2H). Lattice spacing (d_1,0_) was not changed by either compound as lengthening significantly decreased lattice spacing in both conditions (Supplemental Figure 2, A and B). Myosin heads became less ordered along the myosin backbone in response to EDG-7500, but not EDG-7499, exposure as reflected by a decrease in the intensity of the M3 (I_M3_; Supplemental Figure 2, C and D), M6 (I_M6_; Supplemental Figure 2, E and F), and MLL1 (I_MLL1_; Supplemental Figure 2, G and H) meridional reflections.

### Acute EDG-7500 preserves normal cardiac reserve and displays lesser ventricular functional inhibition compared with EDG-7499.

Sprague Dawley rats were used to demonstrate translation of compound potency and force inhibition to in vivo cardiac function measured by echocardiography. WT rats were orally dosed with either EDG-7500 (at 3, 10, 30, and 60 mg/kg) or EDG-7499 (at 0.3, 1.0, and 3.0 mg/kg) with dose estimated by in vitro potency. Echocardiography was performed on ventricular dimensions at 1, 4, 7, and 24 hours after the dose to explore pharmacokinetic/pharmacodynamic (PK/PD) relationships. Decreases in FS% following EDG-7500 dosing across a wide exposure range were significantly limited compared with EDG-7499 reflected by a plateau effect at –29.45% (EDG-7500) versus –96.57% (EDG-7499; *P* < 0.001, nonlinear fit of FS% versus plasma concentration) and a significant shift in PK/PD relationship (measured as EC_50_; Figure 2I). These data align with ATPase and muscle fiber data, showing the decreased systolic calcium potency of EDG-7500 limits systolic inhibition compared with EDG-7499.

We also examined the ability to recruit cardiac reserve in response to β-adrenergic agonism under experimental conditions of compound-induced systolic inhibition measuring a decrease of ~30%–40% in FS% for both compounds (Figure 2J). A significant interaction was observed (group X dobutamine dose), indicating the increase in FS% in response to β-adrenergic agonism is compound dependent. Two hours after the dose, inhibition of FS% was significant for both EDG-7500 and EDG-7499 compared with vehicle control (CON) and was not different between dosed groups. In EDG-7500–treated animals, dobutamine recruited FS% back to the level of vehicle CON-treated rats at dobutamine doses ≥ 5 μg/kg/min with full recovery of FS% reserve at 10 μg/kg/min. In contrast, rats dosed with EDG-7499 were limited in their ability to recruit cardiac reserve, with FS% decreased compared with vehicle CON at all experimental data points.

### Acute EDG-7500 improves relaxation without altering force in engineered heart tissue with a myosin heavy chain R403Q mutation.

**Figure 3:**
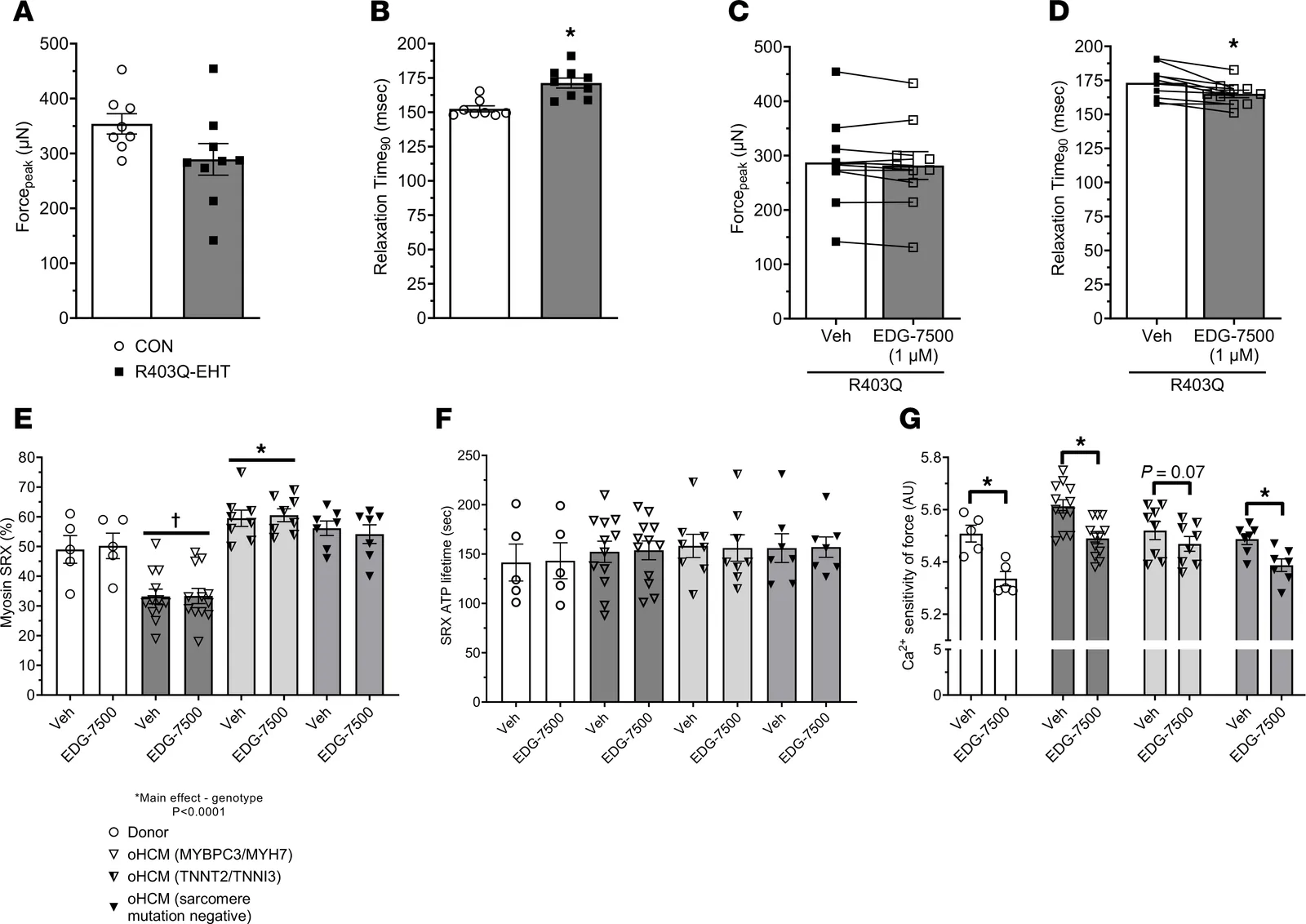
Acute EDG-7500 accelerates relaxation and decreases the Ca^2+^ sensitivity of force without impairing peak force or altering the SRX state in engineered heart tissue with a myosin heavy chain R403Q mutation and human HCM cardiac strips. (**A** and **B**) R403Q-EHT shows a phenotype relevant to HCM with no change in peak force (**A**) and an increase in relaxation time (**B**). (**C** and **D**) Acute exposure to EDG-7500 does not alter peak force but decreases relaxation time in R403Q-EHT, indicating improved lusitropy without altering force. (**E** and **F**) Mant-ATP chase experiments in human cardiac strips show EDG-7500 does not alter the percentage of myosin heads in the super-relaxed (SRX) state (**E**) or the ATP lifetime for molecules in the SRX state (**F**). A main effect of mutation (**E**; *P* < 0.0001, 2×2 ANOVA) showed SRX state decreased in patients with myosin mutations (MYBPC3/MYH7; ^†^*P* < 0.05 versus all genotype groups) and increased in patients with thin filament mutations (TNNT2/TNNI3; *P < 0.05 versus donor and myosin mutation groups). (**G**) In human cardiac strips, the Ca^2+^ sensitivity of force showed a significant decrease after EDG-7500 exposure in almost all groups with a strong trend toward significance in patients with TNNI3/TNNT2 mutations (*P < 0.05, 2×2 ANOVA; main effect of EDG-7500).

Utilizing engineered heart tissue (EHT) technology, EDG-7500 was tested on a genetic background specific to the R403Q mutation in the myosin heavy chain 7 (*MYH7*) gene, a well-characterized cause of HCM in humans. The R403Q mutation did not change peak force compared with isogenic CON at a pacing frequency of 1 Hz (Figure 3A). However, relaxation time (measured as time to 90% relaxation; RT_90_) was significantly increased in the R403Q EHTs compared with isogenic CON indicating the presence of diastolic dysfunction (Figure 3B). Together, these results demonstrate an HCM phenotype in R403Q EHTs relevant for testing EDG-7500 in an experimental context of disease. EDG-7500 did not change peak force (Figure 3C) but did significantly decrease relaxation time (Figure 3D) in R403Q EHTs, demonstrating its ability to improve relaxation without impacting peak force generation in engineered cells with a clinically relevant HCM mutation.

### Acute EDG-7500 decreases Ca^2+^ sensitivity of force but not SRX state in human cardiac strips.

Loaded methylanthraniloyl-ATP (Mant-ATP) chase experiments on thin cardiac strips (approximately 50 μm wide, 1 mm long; sarcomere length set to 2.0 μm; 8–10 strips per patient/donor) from human HCM donors (Supplemental Table 1) were exposed to EDG-7500 to estimate of the percentage of myosin heads in their biochemical ATP-saving super-relaxed (SRX) state. EDG-7500 (10 μM) had no effect on SRX state (Figure 3E) or SRX ATP turnover time in either patients or donors (Figure 3F). A main effect of mutation was observed (Figure 3E; *P* < 0.05, 2x2 ANOVA) indicating a decrease in SRX state in patients with myosin mutations (MYBPC3/MYH7), while SRX state increased in patients with thin filament mutations (TNNT2/TNNI3), similar to previous reports (16–18). Contractile measurements on separate cardiac strips were used to determine the Ca^2+^ sensitivity of force (19), which showed a significant decrease (*P* < 0.05, 2x2 ANOVA; main effect of EDG-7500) in almost all groups with a strong trend toward significance in sarcomere mutation positive patients with TNNT2/TNNI3 mutations (Figure 3G). These data complement preclinical observations of decreased Ca^2+^ dependence of tension presented in Figure 2B.

### Chronic EDG-7500 treatment improves cardiac function and remodeling in the R403Q pig.

**Figure 4:**
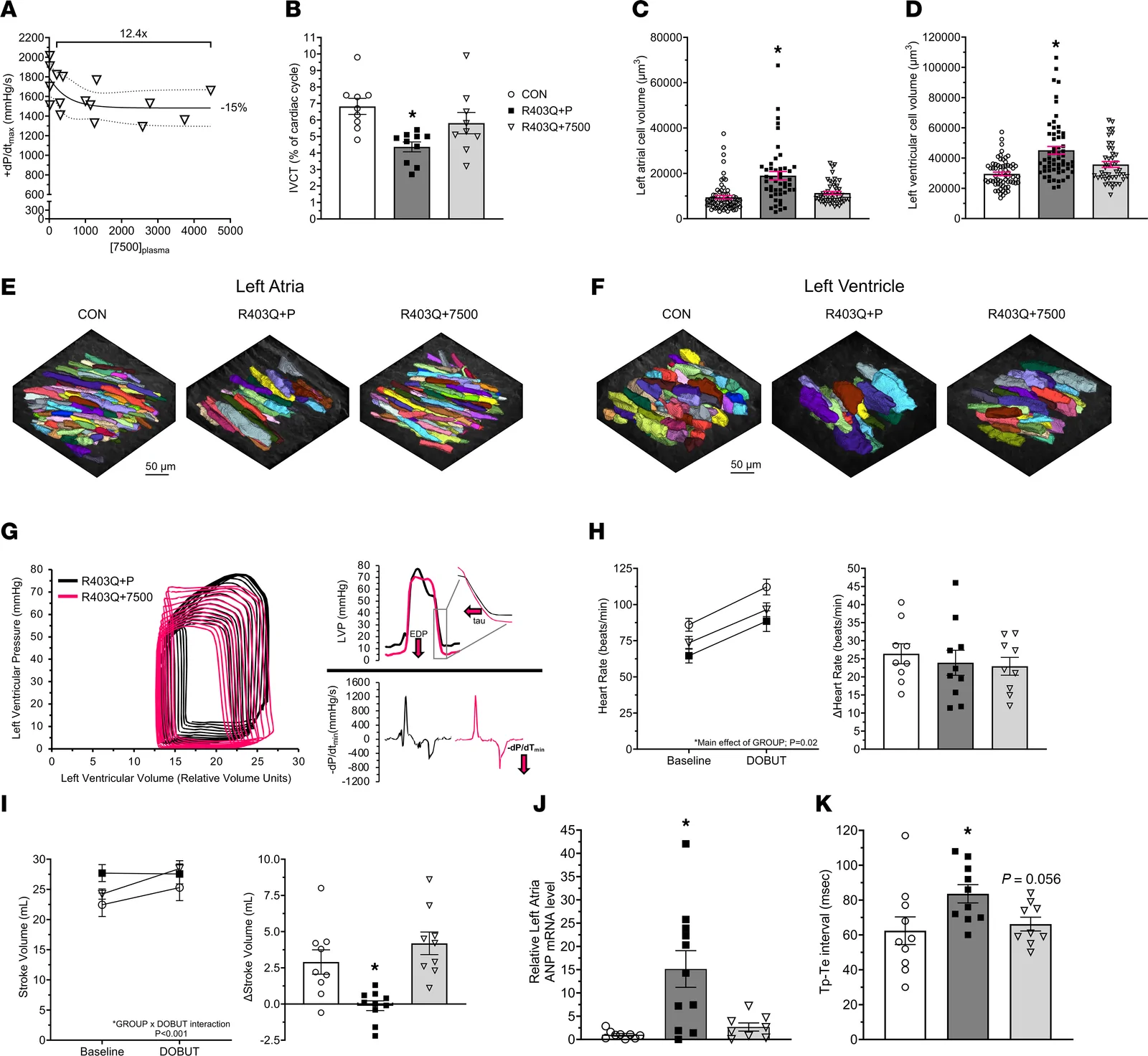
Acute and chronic EDG-7500 slows early LV time to pressure development and systolic ejection while preserving cardiac reserve and preventing molecular and electrical signatures consistent with advancing cardiovascular disease in large animal experimental models of health and disease. (**A**) Systolic LV pressure development (+dP/dt_max_) is decreased initially and then plateaus in a self-limiting manner over a range of increasing plasma levels in response to acute i.v. delivery of EDG-7500 in healthy dogs. (**B**) HCM-mediated decreases in isovolumic contraction time (IVCT) are normalized by EDG-7500 in swine. (**C**–**F**) Increased left atrial (**C**) and ventricular (**D**) pig cardiomyocyte volumes in R403Q+P (*n* = 7; LA regions of interest [ROIs] = 47; LV ROIs = 59) compared with CON (*n* = 9; LA [ROIs] = 69; LV ROIs = 65) are prevented by EDG-7500 (*n* = 6; LA ROIs = 47; LV ROIs = 43). Representative 3D reconstructions of segmented cardiomyocytes from left atrial (**E**) and left ventricular (**F**) pig tissues are presented from one ROI per group. (**G**) Representative pressure-volume loops and hemodynamic traces at rest from individual R403Q+P and R403Q+7500 pigs. (**H** and **I**) Cardiac reserve is preserved through increases in both heart rate (**H**) and stroke volume (**I**) in R403Q+7500 pigs, with Δ responses to β-adrenergic stimulation presented alongside resting Baseline to dobutamine (DOBUT) changes. (**J**) Increased left atrial natriuretic peptide mRNA levels in R403Q+P pigs are prevented by EDG-7500. (**K**) Increased Tp-Te interval in R403Q+P pigs, an electrical marker of impaired LV repolarization, trended toward normalization in R403Q+7500 pigs (*P* = 0.056 versus R403Q+P). *P < 0.05, 1-way ANOVA versus CON and R403Q+7500.

Table 1 and Figure 4 provide functional and structural evidence that EDG-7500 slowed the rate of LV contraction in a self-limiting manner, did not decrease EF%, and prevented LV diastolic impairment in the EDG-7500–treated R403Q (R403Q+7500) group using cMRI, invasive hemodynamic, pressure-volume, postmortem morphology, and molecular techniques. Physiological in vivo target engagement was also demonstrated in healthy instrumented dogs (Figure 4A), whereby the rate of systolic LV pressure development (+dP/dt_max_) showed a self-limiting decrease that plateaus over a range of increasing plasma levels in response to acute I.V. delivery of EDG-7500. Similarly in swine, the decreased isovolumic contraction (IVCT) relative to total cardiac cycle time observed in placebo-treated R403Q (R403Q+P) compared with CON animals was prevented by chronic EDG-7500 treatment in the R403Q+7500 group (Figure 4B). The decrease in relative contraction time was associated with a shift in diastolic filling as a percent of the cardiac cycle in R403Q+P animals, who showed 57% of cycle time spent in diastole versus 43% in systole (577 ± 58 msec versus 403 ± 2 msec, respectively) compared with CON that spent 49% in diastole and 51% in systole (357 ± 30 msec versus 359 ± 9 msec, respectively). EDG-7500 prevented this shift, with the R403Q+7500 group showing 52% of cycle time spent in diastole versus 48% in systole (444 ± 41 msec versus 396 ± 12 msec, respectively), similar to CON animals. Absolute contraction time was the same between all groups (1-way ANOVA, *P* = 0.21; CON = 48 ± 2 msec, R403Q+P = 42 ± 3 msec, R403Q+7500 = 46 ± 3 msec). Together, these results demonstrate the acute and chronic functional effect of EDG-7500 on systole in large-animal experimental models of health and disease characterized by a slowing of LV time to pressure development and the prevention of increased diastolic filling time as a percent of the cardiac cycle.

Body weight and body surface area were the same between groups; thus, absolute heart weight was used for group morphological analyses (Table 1) (20). Significant cardiac remodeling was present in R403Q+P compared with CON animals indicated by an increase in total heart, LV, and left atria weight. Chronic EDG-7500 therapy completely prevented or attenuated pathologic increases in total heart, LV, and LA weight in the R403Q+7500 group. Whole-heart morphology was complemented by similar findings at the cellular level, which showed increases in LA (Figure 4C) and LV (Figure 4D) cardiomyocyte volume in R403Q+P animals compared with CON were prevented by EDG-7500 (Figure 4, E and F). No differences in LV end systolic or diastolic volume were observed between groups, and assessment of peripheral hemodynamics indicated mean arterial pressure was the same for all animals (Table 1).

EDG-7500 did not impair resting LV systolic function and preserved cardiac reserve as assessed by cMRI and pressure-volume hemodynamics. LV EF%, cardiac output, and stroke volume were the same between all groups (Table 1). However, LV contractility (measured as the end systolic pressure-volume relationship [ESPVR]) was increased in R403Q+P compared with CON animals (representative P-V loops and waveform traces are shown in Figure 4G), a finding that was attenuated by EDG-7500 treatment in the R403Q+7500 group (Table 1). Figure 3, H and I, illustrate via pressure-volume hemodynamics that EDG-7500 therapy prevents the loss of cardiac reserve as assessed by dobutamine challenge. Heart rate reserve was unaffected (Figure 4H), although a main effect of decreased heart rate was observed in R403Q+P compared with CON animals (Table 1). However, chronic EDG-7500 therapy preserved normal stroke volume reserve highlighted by a significant interaction indicating that increases in stroke volume (in response to dobutamine) were dependent upon which group the animals were in. Specifically, stroke volume significantly increased in CON and R403Q+7500 animals compared with the R403Q+P group, which showed a complete loss of cardiac reserve in response to β-adrenergic agonism (Figure 4I). These combined data suggest that, although traditional indicators of systolic function like EF% appear normal, an increase in resting LV contractility and lack of cardiac reserve highlight an HCM phenotype in R403Q+P animals that is largely prevented by EDG-7500 therapy.

Chronic EDG-7500 therapy also prevented the diastolic dysfunction observed in the R403Q+P group. Pressure-volume hemodynamics show compliance was significantly impaired in R403Q+P animals compared with CON and evident as an: (a) increase in LV end diastolic pressure; (b) increase in the slope of the end diastolic pressure-volume relationship (EDPVR); (c) decrease in –dP/dt_min_; and (d) increase in Tau (Table 1). These hemodynamic parameters were the same in the R403Q+7500 group compared with CON, demonstrating chronic EDG-7500 therapy completely prevented HCM-mediated diastolic impairment. An increase in cMRI in global native T1-time was also observed in R403Q+P animals compared with the CON and R403Q+7500 groups, indicating chronic EDG-7500 therapy prevented pathologic LV remodeling associated with HCM (Table 1). Evidence for the association of decreased LV compliance and pathologic cardiac remodeling are highlighted by positive correlations between LV end diastolic pressure and both cMRI global native T1-time (Supplemental Figure 3A) and postmortem atrial weight (Supplemental Figure 3B), whereby EDG-7500 shifts group means leftward along the regression line toward normal CON values in R403Q+7500 animals. In total, these data indicate that chronic EDG-7500 therapy prevents genetic and hemodynamic-driven functional changes consistent with HCM-mediated diastolic dysfunction that are associated with pathologic LV structural remodeling.

Increases in common biomarkers associated with the progression of cardiovascular disease and general cardiac health were also inhibited by chronic EDG-7500 therapy. Increased mRNA levels of LV brain natriuretic peptide (Supplemental Figure 4A) and LA atrial natriuretic peptide (Figure 4J) observed in R403Q+P compared with CON were attenuated in the R403Q+7500 group. The R403Q+P group also showed a shift in myosin heavy chain isoform distribution, characterized by an increase in LA β-myosin heavy chain protein level compared with CON animals (Supplemental Figure 4, B and C). This change, considered potentially pathologic in the atria, was prevented in the R403Q+7500 group. The ECG Tp-Te interval (the distance from the peak of the T wave to the end of the T wave), an electromechanical biomarker of diastolic dysfunction (21), increased in R403Q+P animals compared with CON (Figure 4K). This finding, a signal of impaired ventricular repolarization and increased propensity to arrhythmia and sudden cardiac death, was also prevented by chronic EDG-7500 treatment, evident as a trend toward normalization of the pathologically lengthened Tp-Te interval. Overall, these data suggest that chronic EDG-7500 therapy prevents molecular and electrical signatures consistent with advancing cardiovascular disease in the myocardium of animals with genetic HCM.

## Discussion

This study examined 2 enantiomers that following secondary characterization, identified EDG-7500 as a CSM that limited systolic inhibition and prevented diastolic impairment in experimental settings of HCM. The normal role of the RLC is to regulate force production and Ca^2+^ sensitivity of contraction through multiple cellular mechanisms ranging from phosphorylation to interactions with myosin heavy chain, ELC, and myosin binding protein-C, highlighting numerous possibilities for pleiotropic actions (10, 11, 22). Given these complexities, the functional diversity of RLC modulators (and their mechanistic differences compared with traditional CMIs) is not that surprising. In this regard, a primary differentiating feature between CSMs and CMIs is their effect on myosin head movement.

X-ray diffraction techniques showed both EDG-7500 and EDG-7499 move myosin heads toward the thin filament (increased I_1,1_/I_1,0_), similar to RLC-1 (11). This contrasts CMIs like mavacamten, which moves myosin heads toward the thick filament and increases the OFF state (23–25), and aficamten which does not alter I_1,1_/I_1,0_ (26). Differences in x-ray diffraction findings among CMIs and RLC-modulators suggest that small molecule–linked myosin structural movement can be uncoupled from actomyosin ATPase activity, given all of these compounds inhibit actomyosin activity to varying degrees in either calcium-sensitive or calcium-independent ways. Further interrogation highlighted the functional diversity of RLC modulators, demonstrating that EDG-7500 preserves the movement of myosin heads toward the thin filament in response to stretch, whereas heads moved back toward the myosin backbone after stretch in fibers treated with EDG-7499. Consistent with increased intensity ratio (I_1,1_/I_1,0_) and similar to changes caused by stretch (24, 25), myosin heads became less ordered along the myosin backbone in response to EDG-7500. In contrast, EDG-7499 did not change myosin head order at either SL length. The differential effects EDG-7500 and EDG-7499 on I_M3_, I_M6_, and I_MLL1_ meridional reflections and intensity ratio likely influences myosin head recruitment and may play a role regarding the preservation of length-dependent activation and cardiac reserve observed after EDG-7500 exposure. Changes to intensity ratio and myosin head order in fibers treated with EDG-7500 or EDG-7499 order also diverge from observed responses to CMIs. Mavacamten studies show an opposite profile, decreasing I_1,1_/I_1,0_ while increasing I_M3_ and I_MLL1_ meridional reflections (23–25). In contrast to EDG-7499, mavacamten does move heads away from the myosin backbone following stretch with the caveat that I_1,1_/I_1,0_ values only return to normal resting levels (24, 25). Aficamten presents an interesting case in which structural ON/OFF state changes are decoupled from myosin head ordering, showing a smaller reduction in myosin crown ordering than observed in this study or following RLC-1 exposure (11) without altering I_1,1_/I_1,0_ (26). In total, these data reveal differences between RLC modulators and CMIs regarding a defining molecular mechanism of action.

Our data indicate that an important mechanistic difference between d- and s-CSMs is the level of Ca^2+^ sensitivity for ATPase inhibition and force development, indicating that EDG-7500 is primarily active during diastole. Given the enhanced potency of EDG-7499, concentrations for each compound were matched for similar levels of systolic inhibition where appropriate. The inhibitory effects of EDG-7500 on myofibril ATPase activity and Ca^2+^ sensitivity of force (observed in both skinned pig fibers and human donor cardiac strips) were primarily observed at lower Ca^2+^ levels. In contrast, EDG-7499 displayed similar levels of inhibition on myofibril ATPase activity irrespective of Ca^2+^ level without shifting the force-pCa curve, similar to previous reports in cardiac fibers exposed to the CMI mavacamten (27–29). A primary mechanism of RLC-mediated sarcomere regulation is to increase force production and Ca^2+^ sensitivity of contraction via posttranslational modification by MLCK, a calcium- and calmodulin-dependent kinase (10). This mechanism implies that any disease state or compound effect that influences intracellular Ca^2+^ handling may alter the primary regulatory effect of the RLC on myosin and overall sarcomere function. In this regard, the myosin inhibitor RLC-1 decreased baseline and maximal Fura-2 Ca^2+^ fluorescence that was associated with decreased maximal Ca^2+^-activated force and Ca^2+^ sensitivity of force at maximal and submaximal Ca^2+^ levels (11). Together, these data describe differences in Ca^2+^ sensitivity between EDG-7500 compared with EDG-7499 and RLC-1 that potentially influence diastolic- versus systolic-selective properties between RLC-modulators and CMIs.

In EDG-7500–treated fibers, concentration-dependent acceleration of relaxation occurs prior to decreases in activation, highlighting another differentiating feature between RLC modulators. EDG-7500 preferentially affected K_REL_ despite decreased potency compared with EDG-7499, demonstrating again that a mechanistic reliance on the RLC regarding myosin ATPase inhibition does not guarantee similar biophysical phenotypes. The myosin inhibitor RLC-1 also showed a dual ability to both slow the contraction of cardiac fibers and speed their relaxation (11). However, RLC-1 was examined at only 1 concentration (10 μM) that demonstrated relaxation and contraction occurred simultaneously and showed similar effects at 2 separate Ca^2+^ concentrations (i.e., were Ca^2+^ independent) (11). The more in-depth examination of this concept in the current study illustrates that fiber relaxation and activation rates may be concentration dependent, with individual RLC-modulators expressing preferential efficacy that is diastolic- or systolic-selective.

The biochemical and biophysical findings outlined above suggest that d- and s-CSMs might translate to distinct cardiovascular phenotypes in vivo. Examined initially by rat echocardiography, EDG-7500 demonstrated a shallow PK/PD relationship compared with EDG-7499 characterized by reduced systolic inhibition. The steep PK/PD relationship for LV FS% observed in animals exposed to EDG-7499 was similar to that observed in rats treated with traditional CMIs including mavacamten, aficamten, and ulacamten (i.e., CK-586) (28, 30, 31). These findings were comparable with permeabilized muscle results showing more limited inhibition of maximal force in EDG-7500–treated fibers compared with EDG-7499.

Normal systolic reserve was preserved only by EDG-7500, an important finding given the lack of cardiac reserve is a defining feature of HCM and heart failure. When faced with a similar level of compound-induced inhibition prior to stress, EDG-7500–treated animals recovered 100% of normal FS% reserve in response to β-adrenergic agonism. In contrast, rats treated with EDG-7499 saw a significant downward shift compared with vehicle CON. These results confirmed biophysical data showing length-dependent increases in fiber tension and increased I_1,1_/I_1,0_ (surrogates for Frank-Starling) in fibers exposed to EDG-7500. The lack of length-dependent activation in EDG-7499–treated fibers is similar to that observed following exposure to mavacamten (25), further emphasizing differences between RLC modulators and CMIs that are potentially important when considering recovery of cardiac reserve in HCM.

The data distinguishing these enantiomeric pairs provided the rationale to specifically explore EDG-7500 therapeutically in experimental settings of HCM. We focused on the R403Q mutation in the *MYH7* gene, a prominent mutation that causes HCM (32) and promotes disease through concentric cardiac remodeling resulting in cardiac hypertrophy, fibrosis, diastolic dysfunction, and hypercontractility (15). The heterozygous R403Q mutation was assessed in EHT (33) and a Yucatan mini-pig model (12–14). Additionally, we examined human cardiac strips from donors and patients with HCM to determine if the effects of EDG-7500 were specific to myosin, troponin, myosin binding protein-C, or sarcomere mutation–negative samples.

EDG-7500 accelerated relaxation without decreasing peak force in EHTs containing the R403Q mutation and in human cardiac strips, despite not altering the myosin SRX state. While it has been assumed that myosin heads in SRX/DRX states determined by ATP turnover assays are equivalent to OFF/ON states determined by x-ray diffraction (respectively), recent evidence has demonstrated that biochemically defined SRX/DRX can be uncoupled from structural ON/OFF states (34). While the movement of myosin heads into a structurally disordered ON state as measured by x-ray diffraction in Figure 2G may at first appear contradictory, our x-ray diffraction and biochemical data do complement one another in that neither show evidence of increasing the biochemical SRX or structural OFF state. Additionally, the finding of decreased Ca^2+^ sensitivity of force independent of mutation type suggests these biophysical findings were not limited to R403Q-mediated HCM. These observations contrast recently published data, showing ATP consumption associated with the cardiac myosin SRX state in response to the CMI mavacamten is dependent on the myofilament gene variant (*MYL2* versus *TNNI3/TNNT2*) (16). Importantly, these data matched our pCa-tension curves from healthy pigs, indicating the potential for clinical translation.

In R403Q swine, 6 months of chronic EDG-7500 treatment resulted in the normalization of LV filling pressure and drastically improved diastolic compliance (assessed by gold standard cardiac MRI and pressure-volume metrics) that was associated with the prevention of pathological cardiac remodeling in the presence of existing genetic mutation. Significant decreases in native T1 signal and LV/LA size were observed at both the whole heart and cellular level and positively correlated to end diastolic pressure. Pathological increases in atrial size are characteristic of HCM and linked to increased risk of atrial fibrillation (35), suggesting the prevention of LA hypertrophy in EDG-7500–treated animals could reduce the risk of developing this pervasive arrhythmia. Molecular and electromechanical biomarkers reflective of diastolic impairment observed in R403Q+P pigs, including increased LV and LA natriuretic peptide mRNA and the ECG Tp-Te interval (21, 36), were also completely prevented by chronic EDG-7500 therapy.

The prevention of diastolic dysfunction by EDG-7500 was not associated with systolic deficit, suggesting d-CSMs may uncouple diastolic efficacy from systolic impairment to optimize functional benefit. This critical finding illustrates a distinct contrast compared with traditional CMIs like mavacamten and aficamten, which showed that LVOT relief was associated with decreased EF% in felines with genetic HCM (37, 38). In this study, cardiac reserve was preserved in EDG-7500–treated R403Q animals to the same level as that observed in CON swine following β-adrenergic agonism. Together, these results demonstrate EDG-7500 successfully prevented the development of diastolic impairment while preserving normal systolic function and cardiac reserve in a preclinical swine model of HCM with a translationally relevant *MYH7* R403Q mutation.

Important considerations regarding the functional effect of RLC-modulators on the heart include differences in both myosin heavy chain and RLC isoforms between the atria and ventricle (39) and alterations to RLC orientation resulting from differences in temperature (40). In human adult ventricular myocardium, β-myosin heavy chain encoded by the *MYH7* gene and myosin RLC encoded by the *MYL2* gene is predominantly expressed. In the atria, α-myosin heavy chain encoded by the *MYH6* gene and myosin RLC encoded by the *MYL7* gene are most prevalent. These differences can make it difficult to interpret the functional actions of RLC-modulators on a chamber-dependent basis. For example, EDG-7500 prevents the shift from α- to β-myosin heavy chain isoforms in the atria of R403Q pigs (a *MYH7* gene and predominately ventricular mutation; Supplemental Figure 4), suggesting that this change could be related to the significant attenuation of left atrial remodeling following chronic treatment. Additionally, EHT preparations similar to those used in the current study have mRNA levels of both RLC isoforms present (*MYL2* and *MYL7*; data not shown), and temperature-mediated alterations to RLC orientation with respect to the myosin thick filament suggest that myosin head order and recruitment may also be influenced by technical differences in experimental protocols (40). These concepts highlight the need for additional studies to elucidate the mechanistic and chamber-specific actions of RLC-modulators.

In conclusion, we highlight a potentially new class of small molecule that, to the best of our knowledge, displays biochemical, biophysical, and physiological therapeutic profiles contrasting traditional CMIs. Our data demonstrate distinct functional effects between RLC-modulators that predominately benefit diastolic or systolic parameters, leading to potentially novel descriptions we now term d- and s-CSMs. Finally, we showcase EDG-7500 as a d-CSM that uniquely targets both phases of the cardiac cycle by exerting enhanced activity during diastole to directly improve ventricular relaxation while preserving systolic function and cardiac reserve. This dual-phase mechanism differentiates EDG-7500 among both RLC-modulators and CMIs, offering what we believe to be a new therapeutic approach for treating HCM that is currently being investigated in an ongoing multipart Phase 2 trial of participants with oHCM and nHCM (CIRRUS-HCM, NCT06347159).

## Methods

Supplemental Methods are available online with this article.

### Sex as a biological variable and experimental design.

A miniature swine model of nHCM caused by heterozygous *MYH7* R403Q mutation on a Yucatan background (12–14) was used (Exemplar Genetics, Sioux Center, IA). Animals (male, *n* = 16; female, *n* = 15) were randomly assigned into 3 groups: placebo-treated WT CON (*n* = 11: 6 female, 5 male); R403Q+P (*n* = 11: 6 female, 5 male); and R403Q+7500 (*n* = 9: 5 female, 4 male). At 2 months of age, treatment with EDG-7500 (32.5 or 50 mg twice daily, oral; Edgewise Therapeutics) began and continued for 24 weeks. There were no dose or sex-dependent differences; therefore, all data were pooled for analysis.

### Statistics.

Data analyses were performed using Graph Pad Prism version 10.3.1. Group comparisons were made using 2 tailed paired-samples *t* test, 1-way, repeated-measures, or 2-way ANOVA as appropriate. Group differences revealed by ANOVA were found post hoc using Fisher’s LSD multiple comparison test. Regression analyses were used to examine PK/PD and structure-function relationships. All data are presented as mean ± SEM, and significance is reported at the *P* < 0.05 levels.

### Study approval.

Animals were fed once per day, and water was provided ad libitum. All animal protocols were in accordance with the “Principles for the Utilization and Care of Vertebrate Animals Used in Testing Research and Training” and approved by the Edgewise Therapeutics Animal Care and Use Committee. Investigations using human samples conformed to the standards set by the latest version of the Declaration of Helsinki and were run per the approved guidelines of the University of Kentucky (USA)/Erasmus Medical Center (The Netherlands) IRBs (University of Kentucky IRB 46103 and University of Pennsylvania IRB 848421). Informed consent for the research was obtained from the patients or from a legally authorized representative of each donor.

### Data availability.

Underlying data for the manuscript can be accessed in the Supporting Data Values file.

## Author contributions

CAE, MH, MD, SL, CLDR, ME, KK, and AR conceptualized the study. M Michels, along with all the other authors, provided methodology. CAE, MH, MD, SL, L Lee, BB, NAH, M Madden, YW, A Perry, MB, EW, CR, SR, A Peter, ED, SC, JT, SS, CS, WM, SC, JO, DB, DT, JS, CLDR, ME, KK, and AR provided investigation. BLNS and YQ were involved in the design, data collection, and analysis of the x-ray diffraction data presented in Figure 2, G and H. CAE, MH, MD, SL, SR, WM, SC, JO, DT, JS, CLDR, ME, KK, and AR supervised the study. CAE and AJR wrote the original draft of the manuscript. MH, MD, SL, L Lee, NAH, MB, EW, CR, JVDV, CS, WM, L Leinwand, JO, DB, DT, JS, CLDR, MS, ME, and KK reviewed and edited the manuscript.

## Conflict of interest

CAE, MH, MD, SL, LL, BB, NAH, M Madden, YW, ED, SC, JT, SS, CLDR, MS, ME KK, and AR are employees of and own stock or options to purchase stock for Edgewise Therapeutics. A Perry, WM, L Leinwand, SC, JO, and DB consult for Edgewise Therapeutics. WM consults for Cytokinetics Inc. and Kardigan Bio.

## Funding support

- Edgewise Therapeutics.
- BioCAT is supported by grant P30 GM138395 from the National Institute of General Medical Sciences of the National Institutes of Health.

## Supplementary Material

Supplemental data

Unedited blot and gel images

Supporting data values

## Figures and Tables

**Table 1:**
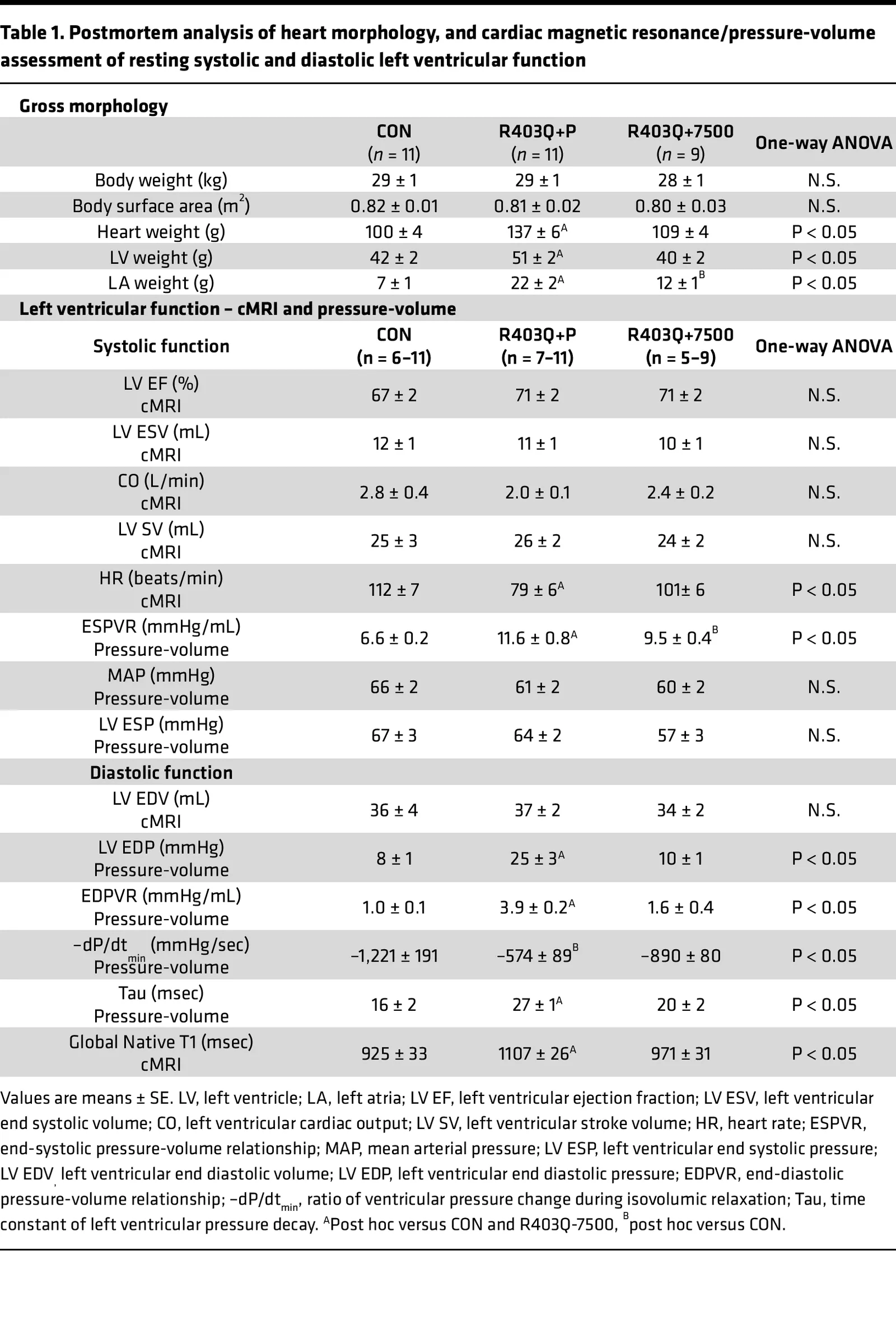
Postmortem analysis of heart morphology, and cardiac magnetic resonance/pressure-volume assessment of resting systolic and diastolic left ventricular function

## Version 1 09/22/2026

Electronic publication

## Version 2 11/09/2026

After publication, the authors became aware that Breanne L. Newell-Stamper and Ying Qian were inadvertently omitted from the author list. They have been included.

## References

[1] Ommen SR (2024). 2024 AHA/ACC/AMSSM/HRS/PACES/SCMR guideline for the management of hypertrophic cardiomyopathy: a report of the American Heart Association/American College of Cardiology joint committee on clinical practice guidelines. Circulation.

[2] Seidman CE, Seidman JG (2011). Identifying sarcomere gene mutations in hypertrophic cardiomyopathy: a personal history. Circ Res.

[3] Desai MY (2025). Long-term favorable cardiac remodeling in obstructive hypertrophic cardiomyopathy patients treated with mavacamten for up to 128 weeks: insights from the VALOR-HCM trial. JACC Cardiovasc Imaging.

[4] Olivotto I (2020). Mavacamten for treatment of symptomatic obstructive hypertrophic cardiomyopathy (EXPLORER-HCM): a randomised, double-blind, placebo-controlled, phase 3 trial. Lancet.

[5] Spertus JA (2021). Mavacamten for treatment of symptomatic obstructive hypertrophic cardiomyopathy (EXPLORER-HCM): health status analysis of a randomised, double-blind, placebo-controlled, phase 3 trial. Lancet.

[6] Garcia-Pavia P (2025). Aficamten or metoprolol monotherapy for obstructive hypertrophic cardiomyopathy. N Engl J Med.

[7] Hegde SM (2025). Effect of aficamten compared with metoprolol on echocardiographic measures in symptomatic obstructive hypertrophic cardiomyopathy: MAPLE-HCM. J Am Coll Cardiol.

[8] Maron MS (2024). Aficamten for symptomatic obstructive hypertrophic cardiomyopathy. N Engl J Med.

[9] Desai MY (2025). Mavacamten in symptomatic nonobstructive hypertrophic cardiomyopathy. N Engl J Med.

[10] Turner KL (2024). The structural and functional effects of myosin regulatory light chain phosphorylation are amplified by increases in sarcomere length and [Ca^2+^]. J Physiol.

[11] Kooiker K (2024). Mechanisms of a novel regulatory light chain-dependent cardiac myosin inhibitor. J Gen Physiol.

[12] Krause AA (2025). Heterozygous MYH7 R403Q mutation impairs left atrial mitochondrial function in a Yucatan mini-pig model of genetic nonobstructive hypertrophic cardiomyopathy. J Appl Physiol (1985).

[13] Sewanan LR (2019). Extracellular matrix from hypertrophic myocardium provokes impaired twitch dynamics in healthy cardiomyocytes. JACC Basic Transl Sci.

[14] Ma W (2022). Myofibril orientation as a metric for characterizing heart disease. Biophys J.

[15] Maron MS, Maron BJ (2013). Hypertrophic cardiomyopathy - authors’ reply. Lancet.

[16] Ochala J (2025). Heterogeneous dysregulation of myosin super-relaxation and energetics in hypertrophic cardiomyopathy. Circ Heart Fail.

[17] Toepfer CN (2020). Myosin sequestration regulates sarcomere function, cardiomyocyte energetics, and metabolism, informing the pathogenesis of hypertrophic cardiomyopathy. Circulation.

[18] Toepfer CN (2019). Hypertrophic cardiomyopathy mutations in MYBPC3 dysregulate myosin. Sci Transl Med.

[19] Ochala J (2023). Predominant myosin superrelaxed state in canine myocardium with naturally occurring dilated cardiomyopathy. Am J Physiol Heart Circ Physiol.

[20] Litwin SE (2015). Cardiac “morphomics”: do we need to measure LV mass and geometry in everyone?. JACC Cardiovasc Imaging.

[21] Sauer A (2012). Diastolic electromechanical coupling: association of the ECG T-peak to T-end interval with echocardiographic markers of diastolic dysfunction. Circ Arrhythm Electrophysiol.

[22] Markandran K (2021). Regulatory light chains in cardiac development and disease. Int J Mol Sci.

[23] Anderson RL (2018). Deciphering the super relaxed state of human β-cardiac myosin and the mode of action of mavacamten from myosin molecules to muscle fibers. Proc Natl Acad Sci U S A.

[24] Ma W (2024). Myosin in autoinhibited off state(s), stabilized by mavacamten, can be recruited in response to inotropic interventions. Proc Natl Acad Sci U S A.

[25] Ma W (2021). The super-relaxed state and length dependent activation in porcine myocardium. Circ Res.

[26] Mohran S (2026). Myosin modulator aficamten inhibits force by altering myosin’s biochemical activity without changing thick filament structure. JACC Basic Transl Sci.

[27] Awinda PO (2021). Mavacamten decreases maximal force and Ca^2+^ sensitivity in the N47K-myosin regulatory light chain mouse model of hypertrophic cardiomyopathy. Am J Physiol Heart Circ Physiol.

[28] Green EM (2016). A small-molecule inhibitor of sarcomere contractility suppresses hypertrophic cardiomyopathy in mice. Science.

[29] Mamidi R (2018). Impact of the myosin modulator mavacamten on force generation and cross-bridge behavior in a murine model of hypercontractility. J Am Heart Assoc.

[30] Hartman JJ (2024). Aficamten is a small-molecule cardiac myosin inhibitor designed to treat hypertrophic cardiomyopathy. Nat Cardiovasc Res.

[31] Rivas VN (2024). Cardiac myosin inhibitor, CK-586, minimally reduces systolic function and ameliorates obstruction in feline hypertrophic cardiomyopathy. Sci Rep.

[32] Geisterfer-Lowrance AA (1990). A molecular basis for familial hypertrophic cardiomyopathy: a beta cardiac myosin heavy chain gene missense mutation. Cell.

[33] Sewanan LR (2021). Mavacamten preserves length-dependent contractility and improves diastolic function in human engineered heart tissue. Am J Physiol Heart Circ Physiol.

[34] Jani VP (2024). The structural OFF and ON states of myosin can be decoupled from the biochemical super- and disordered-relaxed states. PNAS Nexus.

[35] Weissler-Snir A (2024). Atrial fibrillation in hypertrophic cardiomyopathy. JACC Adv.

[36] Panikkath R (2011). Prolonged Tpeak-to-tend interval on the resting ECG is associated with increased risk of sudden cardiac death. Circ Arrhythm Electrophysiol.

[37] Sharpe AN (2023). Effects of Aficamten on cardiac contractility in a feline translational model of hypertrophic cardiomyopathy. Sci Rep.

[38] Stern JA (2016). A small molecule inhibitor of sarcomere contractility acutely relieves left ventricular outflow tract obstruction in feline hypertrophic cardiomyopathy. PLoS One.

[39] Sitbon YH (2020). Insights into myosin regulatory and essential light chains: a focus on their roles in cardiac and skeletal muscle function, development and disease. J Muscle Res Cell Motil.

[40] Park-Holohan SJ (2021). Stress-dependent activation of myosin in the heart requires thin filament activation and thick filament mechanosensing. Proc Natl Acad Sci U S A.

