# Supplementary material for: Mechanistic diversity within RLC-dependent myosin ATPase inhibitors differentiates EDG-7500, a diastolic-selective cardiac sarcomere modulator
Source: JCI Insight. 2026 Sep 22;11(18):e203086. doi: 10.1172/jci.insight.203086 (PMC13596718; doi:10.1172/jci.insight.203086)
Supplement: Supplementary file 1 [file jciinsight-11-203086-s006.pdf]

SUPPLEMENTAL METHODS

Terminal studies - in vivo cardiovascular function. Fasted swine were sedated intramuscular (IM) with Telazol® (6 mg/kg), ketamine (2.2 mg/kg), and acepromazine (0.125 mg/kg), after which peripheral venous access (auricular vein) was secured. Pigs were masked with inhaled isoflurane (~1%) and then orotracheally intubated. Anesthesia was maintained with a continuous fentanyl intravenous (IV) infusion (2.5-5 µg/kg/hr) and isoflurane (1.0-1.6%) in 100% oxygen until completion of the experiment. Pigs were positioned in dorsal recumbence and mechanical ventilation via a volume-cycled animal ventilator was used to sustain end tidal CO_2 values within the physiologic range (35-45mmHg). A 3F micromanometer catheter (Millar Instruments) was inserted into the femoral artery to measure arterial pressure. For left ventricular (LV) mechano-energetic evaluation, a 5F or 7F high-fidelity conductance/micromanometer catheter (Transonic) was advanced retrograde across the aortic valve and into the LV chamber by way of the carotid artery to simultaneously determine left-ventricular pressure and volume. A Swan-Ganz catheter was flow-directed into the pulmonary trunk for cardiac output measurements. A balloon catheter was placed and advanced into the inferior vena cava through the femoral vein. After all catheters were placed, hemodynamic stabilization was allowed for at least 15 minutes prior to the start of the experiments. Pressure-volume (P-V) loops were recorded at rest and following a single dose of dobutamine (to assess cardiac reserve; 2 $\mu g \cdot kg^{-1} \cdot min^{-1}$ iv) under conditions of reducing preload, achieved through transient occlusion of the inferior vena cava via inflation of the balloon catheter. Once steady state was reached (i.e., 5-7 minutes later), CO measurements and a family of pressure-volume curves/loops were obtained. Left ventricular pressure and volume

data were analyzed offline (IOX/ECG Auto; EMKA Technologies) and indices of LV function were generated including: contraction time (as a percent of one cardiac cycle; CT%=100*CT/(60,000/heart rate), heart rate (HR), LV end systolic and diastolic pressure (LVESP and LVEDP, respectively), the end-systolic P-V relationship (ESPVR), and the end-diastolic P-V relationship (EDPVR) (1-3).

Six purpose-bred, 1-2 year old beagle dogs were also examined under similar pressure-volume experimental conditions specifically for assessment of the $+dP/dt_{max}$ PK-PD relationship over a 0.3-3.0 mg/kg dose range. Data were recorded 10 minutes after each dose at steady-hemodynamic state, with selected doses resulted in increasing plasma concentrations of EDG-7500, ranging from 287 ± 30 ng/mL (at 0.3 mg/kg) to 3,210 ± 383 ng/mL (at 3.0 mg/kg). Blood samples were obtained at baseline and following the completion of each dose.

Cardiac magnetic resonance imaging. A subset of Yucatan swine underwent cardiac imaging using a Siemens MAGNETOM Vida 3T MRI scanner (4). Animals were sedated with a cocktail of Telazol (5 mg/kg) / Xylazine (2.2 mg/kg), maintained on 2-3% isoflurane, and ventilated at 6 breaths per minute during ECG-gated cardiac magnetic resonance imaging (cMRI). Physiological parameters, including ECG and SpO2, were continuously monitored throughout the procedure. The cMRI protocol included long-axis cine imaging (2-chamber, 3-chamber, and 4-chamber views), short-axis cine imaging, and pre-contrast T1 mapping performed in both long- and short-axis planes. Cine cMRI sequences were collected in 12-16 parallel, short-axis views at 25 phases/cardiac cycle. Ejection fraction was determined using Simpson method of disks summation to calculate volume and corrected for slice thickness. To calculate LV mass, endo- and epicardial borders of the

LV were contoured manually from short axis cine images at end-diastole and end-systole. The basal slice was included in the analysis if at least 50% of blood volume was surrounded by myocardium. Papillary muscles were excluded from the myocardial mass. The initial contouring of the myocardium was performed using automatic contouring function, with subsequent manual corrections. Left ventricular mass was calculated as left ventricular volume*1.05 g/ ml. Circle Cardiovascular Imaging, Inc. software was used for the analysis (cvi42 version 5.1).

Myofibril Preparation. A separate group of animals provided porcine left ventricle tissue, purchased from Exemplar Genetics (Germantown, MD). Chicken gizzard for smooth muscle myofibrils was purchased from Pel-Freez, LLC (Rogers, AR) (5). Myofibrils were prepared as described previously (6). For ventricle myofibrils, minced tissue was homogenized for 45 seconds with a Polytron homogenizer into 10 volumes (w/v in grams) of Isolation Buffer A (10 mM Imidazole, pH 7.2, 75 mM KCl, 2 mM $MgCl_2$, 2 mM EGTA, 1 mM sodium azide, 1% Triton X-100, supplemented with 4 mM phosphocreatine, 1 mM ATP, 50 mM BDM, 1 mM DTT, 1 mM Benzamidine-HCl, 0.1 mM PMSF, 10 µM leupeptin, 5 µM pepstatin, and 10 mM EDTA). The myofibrils were recovered by centrifugation at 1200 x g for 7 minutes at 4 ºC and resuspended in 10 volumes of Isolation Buffer B (Buffer A without supplemental additions). The myofibrils were homogenized for 45 seconds and recovered by centrifugation at 1200 x g for 7 minutes at 4 ºC. The homogenization and centrifugation step was repeated 3 times with Isolation Buffer B to remove cellular membranes and debris. The myofibrils were then resuspended in Isolation Buffer C (Isolation Buffer B without 1% Triton X-100) and homogenized and collected by

centrifugation as described above. The myofibrils were washed and centrifuged 3 times with Isolation Buffer C before being passed through a 100 μm nylon mesh sheet (Spectrum Laboratories) to remove large particles. The filtered myofibrils were centrifuged at 1200 x g for 15 minutes at 4 ºC and resuspended in 2 to 3 volumes of PM12 buffer (12 mM PIPES, pH 6.8, 2 mM MgCl2, 1 mM DTT). D-sucrose was added to a final concentration of 10% (w/v) and the myofibril suspension was drop-frozen into liquid nitrogen and stored at -80 ºC. Smooth muscle myofibrils were isolated as previously described(5) above with the following changes: Buffer A consisted of 50 mM Tris, 100 mM potassium acetate, 5 mM KCl, 2 mM DTT, 0.1 mM PMSF, 10 μM leupeptin, 5 μM pepstatin, 0.5 mM sodium azide, 5 mM EDTA, and 0.5% Triton X-100, pH 8, Buffer B consisted of 50 mM Tris, 100 mM potassium acetate, 5 mM KCl, 0.5% Triton X-100, and 5 mM EDTA, pH 8, Buffer C consisted of 50 mM Tris, 100 mM potassium acetate, and 5 mM KCl, 2 mM DTT, and 2 mM magnesium acetate, pH 8.0, and the final resuspension buffer consisted of 12 mM PIPES, 2 mM MgCl2, 60 mM KCl, and 1 mM DTT, pH 6.8.

Myosin Full-Length Preparation. Porcine left ventricle full-length myosin was prepared as described in Margossian and Lowey with slight modifications (7). Muscle tissue was passed through a meat grinder rinsed with 0.2 M EDTA and stirred gently for 10 minutes at 4 ºC in 3 volumes (w/v) of extraction buffer (0.3 M KCl, 0.15 M KPi, 20 mM EDTA, 5 mM MgCl2, 2.2 mM ATP, 5 mM DTT, 0.1 mM AEBSF, and 1 protease inhibitor cocktail (Roche), pH 6.7). The extraction was stopped with the addition of 4 volumes of cold water and the homogenate was filtered through cheesecloth to separate the myosin solution from the muscle extract. The myosin solution was then diluted with 6 additional volumes

of cold water and incubated for 2-3 hours at 4 ºC to precipitate the myosin. The precipitated protein was centrifuged at 11,000 x g for 10 minutes at 4 ºC and the resulting pellet was resuspended in a minimal amount of a buffer containing 1 M KCl, 60 mM KPi, 20 mM EDTA, and 5 mM DTT, pH 6.7. This solution was dialyzed overnight at 4 ºC in a buffer containing 0.6 M KCl, 25 mM KPi, 5 mM DTT. The next day, the myosin solution was stirred with an equal volume of cold water and 1 mM ATP for 30 minutes at 4 ºC to precipitate actin-myosin conjugates. The solution was centrifuged at 41,000 x g for 1 hour at 4 ºC, and the supernatant was collected and poured over glass wool to remove any contaminating lipids. The supernatant was then diluted with 9 volumes of cold water and incubated for 2-3 hours to precipitate myosin. The precipitated myosin was centrifuged at 11,000 x g for 10 minutes at 4 ºC, and the resulting pellet was resuspended in 3 M KCl, 50 mM KPi, and 5 mM DTT, pH 6.7. This solution was dialyzed against 0.6 M KCl, 50 mM KPi, and 5 mM DTT, pH 7.0 overnight at 4 ºC. The next day, 1 mM ATP was added before the myosin solution was clarified via centrifugation at 44,000 x g for 2 hours at 4 ºC. The clarified supernatant was poured over glass wool to remove any contaminants. The full-length myosin concentration was determined by a Pierce 660nm Assay (Thermo Fisher), D-sucrose was added to 10% (w/v), and the myosin was drop-frozen in liquid nitrogen and stored at -80 ºC. SDS-PAGE was used to confirm purity of the full-length myosin preparation.

Myosin S1 Fragments Preparation by Proteolytic Digestion. The full-length myosin was papain-digested to myosin subfragment 1 (S1) as previously described with slight modification (7). First, full-length myosin was precipitated at 20 mg/mL in a buffer

containing 20 mM HEPES and 2 mM MgCl2, pH 7.5 and brought to room temperature with slow stirring. Precipitated full-length myosin was incubated with papain (final concentration: 30 μg/mL) in a buffer containing 5 mM cysteine (pH 6.0) and 2 mM EDTA for 7 min at room temperature with gentle stirring. The digest was stopped by rapid addition of 1 mM iodoacetic acid. The digestion was centrifuged at 85,000 x g for 90 min at 4°C and the supernatant was collected. The digested S1 solution was dialyzed overnight against PM12 buffer containing 1 mM DTT, 1 mM ATP, and 10% sucrose and the concentration of myosin S1 was determined using a Pierce 660nm Protein Assay (Thermo Fisher). The purified myosin S1 was flash frozen in liquid nitrogen and stored at -80 °C. SDS-PAGE was used to confirm complete digestion and purity of the S1 fragment.

Actin Purification and Polymerization. Rabbit muscle acetone powder was purchased from Pel-Freez Biologicals and actin was extracted using the method described by Spudich and Watt (8). Acetone powder was extracted for 30 min at 4 °C in Extraction Buffer A (5 mM Tris, 0.2 mM CaCl2, 0.2 mM NaN3, 0.2 mM ATP, and 0.5 mM DTT, pH 8.0). The extracted solution was then filtered through cheesecloth and the tissue was re-extracted in Extraction Buffer A for 20 min at 4°C and filtered through cheesecloth again. The resulting actin supernatants were combined and polymerized with 50 mM KCl and 2 mM MgCl2 with slow stirring for 1 hour at room temperature. Solid KCl was slowly added to a final concentration of 0.8 M and the solution was slowly stirred for 1 hour at 4°C. The polymerized actin was then centrifuged at 142,000 x g for 2 hours at 4 °C and the resulting pellet was resuspended and mechanically homogenized in Extraction Buffer A. The resulting solution was then exhaustively dialyzed against Extraction Buffer A over the next

2-3 days at 4 °C, with 4 buffer changes throughout the dialysis period. The resulting globular actin was then collected from dialysis and centrifuged once more at 142,000 x g for 2 hours at 4°C. The resulting supernatant was collected, and the concentration of globular actin was measured spectrophotometrically at 290 nm and using an extinction coefficient of 0.631.1 $mL \cdot mg^{-1} \cdot cm^{-1}$ to calculate concentration (9). G-Actin was polymerized into F-actin by addition of 25 mM KCl and 5 mM MgCl2, mixed immediately, and incubated at room temperature for 1 hour. The production of F-actin was confirmed by gelling of the solution. F-actin was subsequently used immediately or stored at 4 °C for up to 4 weeks.

Recombinant Myosin Light Chain Proteins Preparation. Human RLC was chosen for the biochemistry RLC-swap experiments due to the high sequence homology with swine RLC (96% sequence identity and 99% sequence similarity) and relevance of this compound to treat human disease. Expression and histidine-tag dependent purification of recombinant regulatory light chain proteins followed standard procedures with slight modification (10). Briefly, 500 ng of *MYL2* (human ventricular regulatory light chain; hvRLC) cDNA plasmid (AtumBio) was incubated for 30 min on ice with 50 µL of BL21(DE3) ultra-competent E. coli cells (New England Biolabs). The mixture was heat-shocked at 42 °C for 10 seconds and incubated on ice for 5 minutes. 950 µL of LB was added to the E. coli mixture under standard sterile conditions and shaken at 250 rpm for 1 hour at 37 °C. 50 µL of the E. coli transformation was then plated onto kanamycin-resistant LB agarose plates and incubated at 37 °C overnight. A single bacterial colony was selected from the plate and added to 50 mL of LB broth + 50 µg/mL kanamycin and shaken at 250 rpm overnight at

37 °C. The E. coli culture was then diluted 1:50 in 1 L LB + 50 µg/mL kanamycin and incubated at 37 °C with shaking at 250 rpm with periodic monitoring of the optical density at 600 nm (OD600) (SpectraMax M3, Molecular Dynamics, San Jose, CA). Once the measured OD600 reached 0.6 to 0.8, the culture was placed on ice for 20 minutes. Isopropyl β-D-1-thiogalactopyranoside (IPTG) was added to a final concentration of 0.2 mM to induce recombinant protein production and the culture was shaken for 4 hours at 250 rpm and 37 °C. The culture was centrifuged at 5000 x g for 15 minutes, and the E. coli cell pellets were resuspended in ice-cold lysis buffer (50 mM NaH_2PO_4, pH 8.0, 300 mM NaCl, 10 mM imidazole, 5 mM BME, 1 mg/mL lysozyme, and 1X protease inhibitor cocktail (Roche). The lysate was then freeze/thaw cycled three times between liquid nitrogen and a 37 °C water bath. After the final 37 °C thaw, DNase I was added to the lysate at a final concentration of 10 µg/mL and mixed for 15 min at room temperature. The lysate was centrifuged at 10,000 x g for 15 minutes at 4 °C. The lysate supernatant was sequentially filtered, first through 5 µm, then 2 µm syringe filters and added to a gravity-flow column containing Ni-NTA agarose beads equilibrated in buffer A (50 mM Tris, pH 7.5, 300 mM NaCl, 40 mM imidazole, 1 mM PMSF, and 5 mM BME). The cell lysate and Ni-NTA resin were incubated for 1 hour at 4 °C with gentle mixing for continuous suspension of the resin. The column was placed upright to settle the resin and gravity flow was used to elute the lysate from the column. The resin was washed with 10-column volumes of buffer A and the His-tagged recombinant RLC proteins were eluted from the column with a buffer containing 50 mM Tris pH 7.5, 300 mM NaCl, 250 mM imidazole, 1 mM PMSF, and 5 mM BME. Fractions were determined to contain purified recombinant RLC proteins via SDS-PAGE, pooled, and dialyzed overnight against 1 L of

10 mM imidazole, 20 mM MOPS, 250 mM NaCl, 1 mM DTT, 0.5 mM EGTA, 2 M urea, pH 7.0 at 4°C. The next day, the purified RLC protein was prepared for TEV-cleavage of the his-tag (11). First, protein concentration was measured spectrophotometrically at 280 nm using an extinction coefficient of 0.00447 mL·mg-1·cm-1. Total protein content was calculated in mg using a calculated molecular weight of 20592.2 g/mol for the recombinant his-tagged RLC protein. A ratio of 10:1 was used for recombinant protein (mg): TEV protease (mg) (AtumBio). The recombinant protein and TEV-protease were incubated at 37 °C for 2 hours with intermittent mixing. The mixture was then centrifuged at 5000 x g for 10 minutes to pellet the precipitate and subsequently filtered through a 1.2 µm syringe filter. The resulting supernatant was then injected to an Ni-NTA column (preequilibrated with 10 mM imidazole, 20 mM MOPS, 250 mM NaCl, 1 mM DTT, 0.5 mM EGTA, 2 M urea, pH 7.0) and incubated with the resin for 10 minutes at 4 °C. The flow through was collected into 1-2 mL aliquots and an additional 2 column volumes of 10 mM imidazole, 20 mM MOPS, 250 mM NaCl, 1 mM DTT, 0.5 mM EGTA, 2 M urea, pH 7.0 was added to wash any additional cleaved-RLC product through the column. SDS-PAGE was used to identify fractions that contained the cleaved-RLC product, pooled together, and dialyzed overnight against SEC buffer (20 mM HEPES, 150 mM KCl, and 2 mM MgCl2, pH 7.5) at 4 °C. After dialysis, protein concentration of the dialyzed protein was measured spectrophotometrically at 280 nm using an extinction coefficient of 0.00298 mL·mg-1·cm-1 and a calculated molecular weight of 18658 g/mol for the cleaved RLC product. Finally, 1 mM TCEP was added to the purified protein and the purified protein flash frozen in liquid nitrogen and stored at -80 °C.

RLC-Exchanged Myosin Full-Length Preparation. Tissue-purified porcine full-length myosin was diluted to 1 mg/mL in 500 mM KCl, 6.4 mM K2HPO4, 3.6 mM KH2PO4, 15 mM CDTA, 1% Triton-X, pH 8.5 and rocked for 15 min at room temperature (12). The RLC-stripping reaction was then stopped by the rapid addition of 10-volumes of ice-cold water containing 10 mM DTT to precipitate the full-length myosin, incubated on ice for 1 hour, and centrifuged at 12,000 x g for 10 minutes at 4 °C. The pelleted RLC-stripped full-length myosin was resuspended in a buffer of 400 mM KCl, 50 mM MOPS, pH 7.0, 2 mM MgCl2, 0.1% Triton-X, and 1 mM DTT. Half of the reaction was stored on ice for later functional assessment. The concentration of myosin in the remaining aliquot was assessed using the Pierce 660nm Assay (Thermo Fisher) and purified recombinant RLC was added (10:1 molar ratio, relative to myosin). The binding reaction was incubated for 2 hours at 4 °C with slow rocking. Quenching of the reaction by precipitation of myosin and centrifugation was performed as above, followed by resuspension in PM12 buffer with 300 mM KCl. The RLC-exchanged full length proteins were stored on ice for functional assessment the same day. Raw DMSO rates for intact, stripped, and reconstituted full-length protein were between 5 and 11 ATP/s^{-1}. SDS-PAGE was used to assess the extent of RLC-stripping and exchange in each reaction.

ATPase Assay. ATPase rates were determined using the NADH-coupled ATPase assay system. All experiments used a buffer system consisting of PM12 buffer with 1 mM DTT, 0.1 mg/mL BSA, 0.5 mM NADH, 1.5 mM phosphoenolpyruvate, 0.01% (v/v) Antifoam 204, and 0.6% (v/v) PK/LDH (final reaction contains ≥3.6 U/mL PK and ≥5.4 U/mL LDH). Porcine left ventricle myofibril ATPase reactions were carried out in the presence of 25

mM ATP and enough free calcium (pCa) to give 0% (pCa 7) or 100% (pCa 5.75) activity. For reactions using porcine left ventricle full-length and S1 myosin, the ATP concentration was increased to 1 mM and no supplemental calcium was added. In these assays, F-actin was added to a final concentration of 0.1 mg/mL Dose-dependent compound effects were assessed by adding the EDG compounds at varying concentrations between 100 μM and 2 nM, all with a constant final DMSO concentration of 1% v/v. The reactions were run at a constant temperature of 25 ºC in a SpectraMax M3 plate reader to monitor the change in absorbance at 340 nm over time. Measurements were taken every 90 seconds for 1 hour and 15 minutes to generate raw time-domain absorbance data, which was processed to normalized reaction rates as described below.

ATPase Assay Data Processing. All data processing was performed using a custom-designed script in Python, coded at Edgewise Therapeutics. Briefly, time-domain absorbance data were first limited to only those points with absorbance between 2.5 AU and a well specific minimum, defined as the greater of 0.5 mAU or any observed absorbance plateau in the well. Trimmed data were then processed with a Savitzy-Golay filter (window size: ~½ of number of datapoints, polynomial: 3) to remove sampling artifacts and reaction rates in mAU/min were then calculated for each well using a linear regression model. Normalized enzymatic rates were calculated, using rates observed in 1% (v/v) in DMSO as 100% and 0 slope as 0%. Compound-specific activity curves were generated by plotting the normalized rates versus compound concentration and fitting the data to a 4-parameter logistic model. In addition to the fit parameters, the IC25 was calculated, relative to the 100% normalized value. The

reported IC25 value is the point of 25% inhibition relative to vehicle control, which was assigned a normalized value of 100. This measurement was chosen as the selected EDG compounds exhibit partial inhibition, resulting in approximately 50% normalized activity even at high concentrations in compound-sensitive systems. DMSO-treated ATPase rates were calculated for each experiment as follows: ventricle myofibrils, pCa 7.0 = 4-7 ATP•s^{-1}; ventricle myofibrils, pCa 5.75 = 9-27 ATP•s^{-1}; ventricle full-length myosin = 4-15 ATP•s^{-1}; ventricle S1 = 6-11 ATP•s^{-1}. Due to differences in raw ATPase rates, data was normalized for each compound to allow for comparable assessment across conditions similar to previous reports (13, 14). Fit parameters calculated effective concentrations, trimmed time-domain absorbance data and calculated slopes were exported, in addition to compound-specific graphs of normalized ATPase activity versus concentration in µM.

Phosphorylation of Smooth Muscle Myofibrils. Smooth muscle myofibrils were phosphorylated using a modified protocol (15). Myofibrils at a final concentration of 3.87 mg/mL were suspended in 10X PM12 buffer (12 mM PIPES, pH 6.8, 2 mM $MgCl_2$). Two reaction conditions were prepared: a negative control, with no myosin light chain kinase (MLCK), and an experimental phosphorylation reaction with MLCK. Both conditions included 10X PM12, 1 µM calmodulin, 5 mM $CaCl_2$, and 5.1 mM ATP. The experimental condition additionally included 9.91µg/mL of MLCK per reaction, while the negative control omitted MLCK. Samples were rotated at 4 °C for approximately 4 hours and then reactions were centrifuged at 3500 rpm for 7 minutes at 4 °C to pellet the myofibrils. Supernatants were carefully discarded without disturbing the pellets and the pellets were

resuspended in 25% of the original reaction volume with 10X PM12. Myofibrils were then mixed via pipetting or passed through a 20 gauge needle to disperse clumps. Protein concentration was assessed using a NanoDrop spectrophotometer at 280 nm using 10X PM12 as a blank. Phosphorylated myofibrils were used immediately in ATPase assays.

Preparation of Cardiac Muscle for Fiber Testing. Cardiac tissue was collected from freshly euthanized wildtype domestic pig hearts stored in cardioplegia solution (Exemplar Genetics, IA, USA). Pig cardiac muscle fiber bundles were dissected from left ventricular muscle wall at 4°C in dissecting solution containing 250 mM K-propionate, 40 mM Imidazole, 10 mM EGTA, 4 mM $MgCl_2$, and 2 mM ATP (Roche, Gumucio et al. 2015). Fiber bundles were chemically skinned with dissecting solution containing 1% Triton X-100 for 30 minutes at 4°C and then transferred into storage solution (1:1 dissecting solution and glycerol) overnight at 4°C before fiber bundles were transferred to fresh storage solution and stored long term at -80°C.

Ex Vivo Testing of Skinned Fibers: On the day of each experiment, a muscle bundle was thawed from the -80°C freezer and placed in relaxing solution in a conical tube. Tissue was then homogenized with a polytron at midpoint speed (12,000 – 17,000 rpm) for 5 – 10 seconds (16). Once homogenized, 1 – 2 mL of tissue slurry was transferred to a 5 cm sterile petri dish containing 5 mL of fresh relaxing solution. A suitable fiber was selected based on dimensions (0.5 – 1.5 mm length by 100 – 200 μm width) and then transferred to the fiber testing apparatus (1400A, Aurora Scientific, Canada) containing fresh relaxing solution. The fiber was draped between two stainless steel pins connected to a force

transducer and motor, and glued in place (Collodion glue, Germany) (17). The average sarcomere length was set to 2.0 µm (short SL) using the VSL 900B program and camera on an inverted light microscope (Olympus IX53, Japan), and 2.3µm for LDA lengthening experiments (long SL).

The bath temperature was set to 22°C using a temperature-controlled thermocouple and Peltier cooling system (Aurora Scientific, Canada). Experimental solutions and protocols were prepared as previously described with minor deviations (16, 18). Relaxing solution contained 100 mM BES, 10 mM EGTA, 6.6 mM $MgCl_2$, 41.9 mM K-propionate, 10 mM Na_2CrP, 6.2 mM ATP, 100 µM A2P5, 1 mM DTT, 2.5 µM pepstatin, 1 µM leupeptin, 50 µM PMSF, and 5 mM NaN_3, pH 7.0. Activating solution contained 100 mM BES, 10 mM EGTA, 6.2 mM $MgCl_2$, 22.2 mM K-propionate, 10 mM Na_2CrP, 6.3 mM ATP, 100 µM A2P5, 1 mM DTT, 2.5 µM pepstatin, 1 µM leupeptin, 50 µM PMSF, 5 mM NaN_3, and 10 mM $CaCl_2$, pH 7.0 All control solutions contained 1% DMSO and test article pCa solutions contained the desired concentration of EDG-7500 or EDG-7499 suspended in DMSO (1% final). Fiber quality was controlled by a minimum baseline force of 0.10 mN required for the fiber to be included in the analysis, and an average maximum specific force under DMSO conditions prior to compound exposure similar to previous reports showing no difference in biophysical parameters between glycerinated and cryopreserved porcine fibers (31.39 ± 5.57 kN•m^{-2}) (19). Solutions were then replaced with the same calcium solution ratios containing compound, and the same fiber was tested again with the same protocol. Each trace was compared between control and compound runs, to evaluate compound effectiveness in altering force, Hill slope and calcium sensitivity. Force was reported as the difference between the DMSO high calcium

solution (maximum force at pCa 4.5) and compound high calcium solution. Dose response EC_{50} was calculated from fitted data using a four-parameter nonlinear regression equation using Graphpad Prism.

For length-dependent activation (LDA) experiments, once the fiber force reached steady state force at a given compound concentration, the fiber was lengthened to 2.3 µm passively. After fiber equilibration for several minutes at 2.3 µm, the equilibration solution was replaced with new solution containing the same compound and concentration, and the same fiber was tested again at this new length. Length-dependent activation of force was reported as the difference between short (2.0 µm) versus long (2.3 µm) fiber length. Control LDA measures were collected from a separate pool of fibers exposed to vehicle (DMSO).

To measure the rate of fiber activation (k_{ACT}) and relaxation (k_{REL}), a stream of relaxing solution was directed at a maximally activated bundles of myofibrils approximately 150 µm in diameter (EDG-7500, 157 ± 6 µm vs. EDG-7499, 147 ± 5 µm; P = 0.17) using a reciprocal syringe pump (flow rate: 250 µL/min; 5 mL syringe capacity). The fiber was primed in DMSO activating solution until force reached steady state (force plateau), at which point the syringe pump containing relaxing solution was started resulting in rapid fiber relaxation. The fiber was then pre-incubated for 10 minutes in relaxing solution with increasing concentrations of compound until 100 µM was reached. The compound concentration ladder was from lowest to highest. Kinetics of skinned fibers are similar to previous reports using bundles of myofibrils ~25-80 µm in size (20, 21).

X-ray Diffraction Experiments: Porcine left ventricular myocardium samples were prepared following the methodology outlined in our previous study (22). Briefly, frozen porcine left ventricle myocardium was defrosted in skinning solution (91 mM K^+-propionate, 3.5 mM MgCl, 0.165 mM $CaCl_2$, 7 mM EGTA, 2.25 mM Na^2ATP, 15 mM sodium creatine phosphate, 20 mM Imidazole, 15 mM BDM, 1% Triton-X100) at room temperature before dissection into small strips aligned along the fiber orientation. The strips were further skinned until the tissue became transparent (2-3 hours). The tissues were then washed with relaxing solution (91mM K^+-propionate, 3.5mM $MgCl_2$, 0.16mM $CaCl_2$, 7mM EGTA, 2.25 mM Na^2ATP, 15 mM creatine phosphate, 20 mM Imidazole at pH 7) three times, 10min each. Well-aligned tissues were further dissected into preparations with a length of 4mm and a diameter of 200-300 µm before being attached to aluminum T-clips at both ends. The clipped preparations were stored in cold relaxing solution containing 3% dextran at 4°C for use in experiments on the same day.

X-ray diffraction experiments were performed at the BioCAT beamline 18ID at the Advanced Photon Source, Argonne National Laboratory, and the BL4-2 beamline at Stanford Synchrotron Radiation Lightsource, SLAC National Accelerator Laboratory. The X-ray beam energy was set to 12 keV (0.1033 nm wavelength) at an incident flux of ~10^{12} photons per second. The specimen-to-detector distance was approximately 3 m. The preparation was then attached to a hook on a force transducer (Model 402B, Aurora Scientific Inc., Aurora, ON, Canada) and a static hook. The muscle was incubated in a customized chamber with Kapton windows on the X-ray path. The bottom of the chamber was attached to a heat-exchanger so the solution was kept between 28 °C and 30 °C. The sarcomere length was adjusted by using micromanipulators attached to the hooks

while monitoring the light diffraction pattern from a helium-neon laser (633 nm) on a screen. The X-ray pattern was collected using a MarCCD 165 detector at BioCAT and a Rayonix MX225-HE CCD detector at BL4-2 (Rayonix Inc., Evanston, IL), with a 1-second exposure time. Two to three X-ray diffraction patterns were collected under each condition. To minimize radiation damage, the muscle was oscillated along its horizontal axes at a velocity of 10 mm/s. The beam positions on the samples were moved vertically after each exposure to avoid overlapping X-ray exposure.

Data were analyzed using data reduction programs belonging to the open-source MuscleX software package developed at BioCAT (23). The equatorial reflections were measured by the “Equator” routine in MuscleX. The equatorial intensity trace was reduced to a one-dimensional projection by drawing a box along the equator with a width set to be just outside the reflections, the 1,0 and 1,1 reflection were modeled on Gaussian function superimposed on a smooth background as described previously (24). For subsequent analyses, the four equivalent quadrants of X-ray images were added together to improve the signal noise ratio with the “Quadrant Folding” routine in MuscleX. The spacing of a targeted reflection was estimated by measuring the distance from the beam center to the centroid of the peak profile considering only the top half of the diffraction peak (25). The intensity of a targeted reflection was calculated by fitting the peak profile to a Gaussian function. To compare the intensities under different conditions, the measured intensities of X-ray reflections were normalized to the sixth-order actin-based layer line intensities as described previously (26). Values obtained from patterns under the same condition were averaged.

In vivo Rat Echocardiography: For all studies, Sprague Dawley rats were weighed and dosed PO with compound via an 18-gauge gavage needle with a dose-volume of 5 mL/kg. Following dosing, echocardiography was performed at 1, 4, 7, and 24 hours post-dose. EDG-7500 was administered at 3, 10, 30, and 60 mg/kg, and EDG-7499 was administered at 0.3, 1.0, and 3.0 mg/kg. Transthoracic echocardiography (TTE) assessment was performed under isoflurane anesthesia using a Vevo2100 ultrasound system with a MX250 probe. Anesthesia was induced at 5% isoflurane for 2-3 minutes, followed by maintenance at 2% for the duration of the study. Temperature of the animals was monitored and maintained through a rectal probe coupled to a bioregulator to maintain 37°C. Surface ECG and respiration were monitored through the same system. The left-ventricular (LV) chamber was imaged using parasternal long axis M-mode images to determine LV dimensions and fractional shortening. Left-ventricular end-systolic dimensions (LVID;s) and left-ventricular end-diastolic dimensions (LVID;d) were measured from M-mode images from the long-axis view. Fractional shortening (FS) was calculated using the following equation FS = ((LVID;d - LVID;s) / LVID;d) * 100. FS is shown as % change vs baseline/vehicle, calculated as: FS (% change from baseline) = FS (post dose) / FS (baseline) * 100.

Cardiac reserve was also assessed in Sprague Dawley rats. Animals were weighed and dosed PO with Vehicle, EDG-7500, or EDG-7499 via an 18-gauge gavage needle with a dose-volume of 5 mL/kg. A FS% reduction of ~40% for each compound was targeted to assess cardiac reserve in the presence of compound-induced inhibition. Animals were dosed with 10-30 mg/kg of EDG-7500 and 1-3 mg/kg EDG-7499. At 2 hrs post vehicle or EDG compound, dobutamine was infused at increasing rates to deliver

respective target dose of 2.5, 5.0, and 10.0 ug/kg/min to determine systolic reserve in response to β-adrenergic agonism.

Engineered Heart Tissue Experiments: The effects of EDG-7500 were determined in human engineered heart tissues (EHTs) formed in MyoPod® scaffolds seeded with ventricular human induced pluripotent stem cell (hiPSC)-derived cardiomyocytes. At testing, the hiPSC-CMs are 43–49 days post-differentiation. Cells were 22–25 days old at EHT formation, after which tissues were cultured an additional 21–24 days before testing. Previous work has shown the myosin isoform in this EHT cell line is >90% β-MYHC (27). The MYH7 R403Q line is a heterozygous mutation. Specifically, cells were differentiated from PGP1 hiPSCs harboring the MYH7 R403Q+/- mutation (R403Q) or PGP1 wild-type isogenic control hiPSCs (Control). Tissues were produced by seeding hiPSC-cardiomyocytes and primary human cardiac fibroblasts (hCFs) in decellularized porcine myocardial scaffolds (6.34 mm x 2.75 mm x 150 μm, LxWxH) at $1x10^6$ hiPSC-cardiomyocytes and $1x10^5$ hCFs per tissue. Engineered heart tissues were cultured for three to four weeks and then tested in the MyoLab®, Propria's automated contractility testing device. For all testing, tissues were mounted in the device at culture length (0% stretch). Control and R403Q tissues were perfused at 37ºC in 5% CO2-equilibrated DMEM +0.01% DMSO at 0.3 mL/min and were paced at 1-1.1 Hz. After mounting, EHTs underwent preconditioning: they were stretched from -5% to 5% of culture length three times. EHTs were then returned to culture length and equilibrated for a total minimum equilibration time of 35 minutes (including mounting and preconditioning). After equilibration, EHTs underwent 6-point dose-response testing from 0.1 to 30 uM EDG-

7500 for effects on peak force and relaxation time. Tissues were paced and perfused continuously throughout every study at 1 Hz. Three, 5-10 second recordings at each condition were averaged to calculate contractile metrics at baseline and at each dose tested.

Human myocardial samples. Investigations using human samples conformed to the standards set by the latest version of the Declaration of Helsinki, and were run per the approved guidelines of the University of Kentucky (USA)/ Erasmus Medical Center (The Netherlands) institutional review boards (University of Kentucky IRB 46103 and University of Pennsylvania IRB 848421). Informed consent for the research was obtained from the patients or from a legally authorized representative of each donor.

Interventricular septal tissue from patients with obstructive HCM was obtained during myectomy surgery at the Erasmus Medical Center (The Netherlands) and immediately snap-frozen and stored in liquid nitrogen. Septal tissue from non-failing donors with no recorded history of cardiac disease was obtained during organ donation at the University of Kentucky (USA). Cardiac donor tissue was also rapidly flash-frozen in liquid nitrogen. All samples were shipped on dry ice to the University of Copenhagen (Denmark) and kept at -80°C until use.

Cardiac strip preparation and permeabilization. The cryopreserved samples were dissected into thin bundles and immersed in a membrane-permeabilising solution (relaxing solution containing glycerol; 50:50 v/v) for 24 hours at -20°C, after which they were transferred to 4°C. These bundles were kept in the membrane-permeabilising

solution at 4°C for an additional 24 hours (to allow a proper skinning/membrane permeabilization process). After these steps, bundles were stored in the same buffer at -20°C for use up to one week (28).

Solutions. As previously published (28), the relaxing solution contained 4 mM Mg-ATP, 1 mM free Mg^{2+}, $10^{-6.00}$ mM free Ca^{2+}, 10 mM imidazole, 7 mM EGTA, 14.5 mM creatine phosphate. Ionic strength was adjusted to 180 mM and pH to 7.0. Additionally, the rigor buffer for Mant-ATP chase experiments contained 120 mM K acetate, 5 mM Mg acetate, 2.5 mM K_2HPO_4, 50 mM MOPS, 2 mM DTT with a pH of 6.8.

Mant-ATP chase experiments. On the day of the experiments, bundles were transferred to the relaxing solution and thin cardiac strips (approximately 50 µm wide and 2 mm long) were isolated. Their ends were individually clamped to half-split copper meshes designed for electron microscopy (SPI G100 2010C-XA, width, 3 mm), which had been glued to glass slides (Academy, 26 x 76 mm, thickness 1.00-1.20 mm). Cover slips were attached to the top using double-sided tape to create flow chambers (Menzel-Gläser, 22 x 22 mm, thickness 0.13-0.16 mm). Subsequently, at 22°C (or 8°C), for each cardiac strip, the sarcomere length was measured using the brightfield mode of a Zeiss Axio Scope A1 microscope, a Zeiss AxioCam ICm 1 camera and a LD Plan-Neofluar 40x/0.60 objective. Preparations with a sarcomere length of 2.00 ± 0.05 µm were further subjected to the following. Similar to previous studies, each thin strip was first incubated for five minutes with a rigor buffer. A solution containing the rigor buffer with 250 µM Mant-ATP was then flushed and kept in the chamber for five minutes. At the end of this step, another solution

made of the rigor buffer with 4 mM ATP was added with simultaneous acquisition of the Mant-ATP chase. For fluorescence acquisition, a Zeiss Axio Scope A1 microscope was used with a Plan-Apochromat 20x/0.8 objective and a Zeiss AxioCam ICm 1 camera. Frames were acquired every five seconds with a 20 ms exposure time using a DAPI filter set, for 5 min. Tests were run prior to starting the current study where images were collected for 15 min instead of 5 min – these tests did not reveal any significant difference in the parameters calculated. Three regions of each individual strip were sampled for fluorescence decay using the ROI manager in ImageJ as previously published (28). The mean background fluorescence intensity was subtracted from the average of the fiber fluorescence intensity (for each image taken). Each time point was then normalized by the fluorescence intensity of the final Mant-ATP image before washout (T = 0). These data were then fit to an unconstrained double exponential decay using Graphpad Prism 10.4:

$$Normalised\ Fluorescence = 1 - P1\left(1 - exp^{(-t/T_1)}\right) - P2\left(1 - exp^{(-t/T_2)}\right)$$

Where P1 is the amplitude of the initial rapid decay approximating the ATP-consuming disordered-relaxed state with T1 as the time constant for this decay. P2 is the slower second decay approximating the proportion of myosin heads in the ATP-conserving super-relaxed state with its associated time constant T2. In the present article, P2 and T2 are shown in the results section.

Ca²⁺ sensitivity of force. As for the Mant-ATP chase experiments, on the day of the experiments, bundles were transferred to the relaxing solution and thin cardiac strips were dissected. They were then individually mounted between connectors leading to a force transducer and a lever arm using the MechaMorph system (29). Sarcomere length was set to 2.00 ± 0.05 μm and the temperature to 15°C(28). Cardiac strips were subsequently activated in solutions containing increasing levels of free Ca^{2+} as previously published(29).

Tissue preparation and immunohistochemistry for LA/LV cell volume assessment. Cardiac tissue was processed for thick-section immunohistochemistry as previously described (30). Briefly, porcine cardiac tissues from left ventricle and atria were fixed en bloc in 4% paraformaldehyde, cryoprotected in sucrose, and embedded in OCT before sectioning at 150 μm thickness. Free-floating sections were permeabilized and stained with wheat germ agglutinin (WGA, Alexa Fluor 488; Thermo Fisher, W11261) to delineate extracellular boundaries and with mouse anti–α-actinin conjugated to Alexa Fluor 647 (Sigma-Aldrich, A7732) to identify cardiomyocytes. Sections were mounted with SlowFade Glass mountant (Thermo Fisher, S36917) under #1.5 cover glass prior to imaging.

Confocal imaging. Samples were imaged using a Yokogawa CSU-W1 spinning disk confocal microscope coupled to a Nikon Ti2 inverted stand. Multi-channel scout images were acquired at 20x magnification to identify representative regions of interest (ROIs). For each specimen, 6–10 ROIs were selected in regions containing longitudinally or

obliquely oriented cardiomyocytes while avoiding perivascular or extensively fibrotic areas. High-resolution z-stacks were then acquired at 40x oil immersion with voxel dimensions of 0.162 x 0.162 x 1 µm, covering 100–150 µm in depth. Images were exported as ND2 files for downstream analysis.

3D cardiomyocyte segmentation and morphometric analysis. Cardiomyocytes were segmented using CaMVIA-3D, a deep-learning pipeline based on a custom-trained Omnipose model optimized for elongated and variably shaped cardiomyocytes, as previously described (30). Briefly, raw ND2 files were preprocessed in FIJI using the CLIJ2 plugin to correct for z-depth intensity attenuation. Channels were resampled and saved as TIFF z-stacks for segmentation. The CaMVIA-3D model was applied to WGA-labeled z-stacks to generate cardiomyocyte masks. Post-processing of segmentations was performed in Napari to remove partial or fragmented cell masks, followed by 1-pixel erosion to refine boundaries. Morphometric features including cell volume were extracted using napari-skimage-regionprops plugin. Summary statistics were computed per cell and ROI for downstream analyses.

*RNA isolation, complementary DNA synthesis, and quantitative PCR***.** Total RNA from cryo-ground pig LV and atria was purified by homogenizing tissue in TRI Reagent (Sigma-Aldrich, T9424) following the manufacturer's protocol. RNA was transcribed to cDNA using the High-Capacity cDNA Reverse Transcription Kit with RNase Inhibitor (Applied Biosystems, 4374966) according to the manufacturer's protocol. qPCR was performed

on a Bio-Rad CFX384/C1000 Touch thermal cycler using SYBR Green PCR Master Mix (Applied Biosystems, 4309155) following the manufacturer's instructions. Target gene expression was analyzed using the $2^{-\Delta\Delta}$Ct method as previously described (31-33) and normalized to 18S. Molecular assessment of samples revealed as statistical outliers, defined as >2 S.D. away from the mean (i.e., outside the 95% confidence interval), were removed from the analysis. Analysis of 18S and brain natriuretic peptide (BNP) mRNA levels was performed using primers previously published (33), and atrial natriuretic peptide (ANP) mRNA levels were measured using the following primers: ANP (F: 5'-GAGCTTCCTCCTCGTTCTGG -3'; R: 5'- CCAAGTGGTCCAGCAAATTCTTGA -3').

Muscle Protein Homogenate Preparation for Myosin Heavy Chain Distribution. Sections of swine left atrial tissue were collected and snap frozen in liquid nitrogen and stored until processed. Cryo-ground tissue was thawed and protein extracted in a buffer containing 8M urea, 2M thiourea, 50mM Tris pH 6.8, 75mM DTT, 3% SDS, 0.05% bromophenol blue over-night at 4°C. Total protein concentration of the clarified supernatant was determined using a Pierce600 assay according to manufacturer's protocol (ThermoScientific). Porcine atrial myofibrils were purified from muscle as described above.

Myosin heavy chain gel preparation and quantification: A myosin separating gel was prepared according to Warren and Greaser (cite PMID: 12895480) with slight modification. Briefly, an 8% resolving gel was prepared for optimal separation of the myosin isoforms in the pig samples. 1µg of total protein lysate was loaded on the gel for each sample and the gel was run at 85V for 28 hours at 4°C. 1µg of porcine ventricular

and atrial myofibrils were run as beta and alpha MHC controls, respectively. Total protein was quantified using the SyproRuby Total Protein stain (rapid protocol followed according to product manual) and visualized on a LiCor Odyssey M. Quantification of α- and β-myosin heavy chain was performed using Empiria Studio 2.2 software (LiCor, Inc.), with expression of each isoform was quantified as a percentage of total myosin heavy chain protein.

Synthesis strategy and chemical characterization of EDG-7499 and EDG-7500: Compound procedures and characterization for EDG-7500/7499 are described in Patent WO2024073426 (34).

REFERENCES

1. Hiemstra JA, Lee DI, Chakir K, Gutierrez-Aguilar M, Marshall KD, Zgoda PJ, et al. Saxagliptin and Tadalafil Differentially Alter Cyclic Guanosine Monophosphate (cGMP) Signaling and Left Ventricular Function in Aortic-Banded Mini-Swine. *J Am Heart Assoc.* 2016;5(4):e003277.
2. Olver TD, Edwards JC, Jurrissen TJ, Veteto AB, Jones JL, Gao C, et al. Western Diet-Fed, Aortic-Banded Ossabaw Swine: A Preclinical Model of Cardio-Metabolic Heart Failure. *JACC Basic Transl Sci.* 2019;4(3):404-21.
3. Olver TD, Edwards JC, Ferguson BS, Hiemstra JA, Thorne PK, Hill MA, et al. Chronic interval exercise training prevents BK(Ca) channel-mediated coronary vascular dysfunction in aortic-banded miniswine. *J Appl Physiol (1985).* 2018;125(1):86-96.
4. Tee MW, Won S, Raman FS, Yi C, Vigneault DM, Davies-Venn C, et al. Regional Strain Analysis with Multidetector CT in a Swine Cardiomyopathy Model: Relationship to Cardiac MR Tagging and Myocardial Fibrosis. *Radiology.* 2015;277(1):88-94.
5. Sobieszek A, and Bremel RD. Preparation and properties of vertebrate smooth-muscle myofibrils and actomyosin. *Eur J Biochem.* 1975;55(1):49-60.
6. Herrmann C, Sleep J, Chaussepied P, Travers F, and Barman T. A structural and kinetic study on myofibrils prevented from shortening by chemical cross-linking. *Biochemistry.* 1993;32(28):7255-63.
7. Margossian SS, and Lowey S. Preparation of myosin and its subfragments from rabbit skeletal muscle. *Methods Enzymol.* 1982;85 Pt B:55-71.
8. Spudich JA, and Watt S. The regulation of rabbit skeletal muscle contraction. I. Biochemical studies of the interaction of the tropomyosin-troponin complex with actin and the proteolytic fragments of myosin. *J Biol Chem.* 1971;246(15):4866-71.
9. Borrego-Diaz E, and Chalovich JM. Kinetics of regulated actin transitions measured by probes on tropomyosin. *Biophys J.* 2010;98(11):2601-9.
10. Loughran ST, and Walls D. Purification of poly-histidine-tagged proteins. *Methods Mol Biol.* 2011;681:311-35.
11. Ceron RH, Carman PJ, Rebowski G, Boczkowska M, Heuckeroth RO, and Dominguez R. A solution to the long-standing problem of actin expression and purification. *Proc Natl Acad Sci U S A.* 2022;119(41):e2209150119.
12. Karabina A, Kazmierczak K, Szczesna-Cordary D, and Moore JR. Myosin regulatory light chain phosphorylation enhances cardiac beta-myosin in vitro motility under load. *Arch Biochem Biophys.* 2015;580:14-21.
13. Hartman JJ, Hwee DT, Robert-Paganin J, Chuang C, Chin ER, Edell S, et al. Aficamten is a small-molecule cardiac myosin inhibitor designed to treat hypertrophic cardiomyopathy. *Nat Cardiovasc Res.* 2024;3(8):1003-16.

14. Kooiker K, Gan QF, Yu M, Sa N, Mohran S, Cheng Y, et al. Mechanisms of a novel regulatory light chain-dependent cardiac myosin inhibitor. *J Gen Physiol.* 2024;156(10).
15. Sellers JR, Spudich JA, and Sheetz MP. Light chain phosphorylation regulates the movement of smooth muscle myosin on actin filaments. *J Cell Biol.* 1985;101(5 Pt 1):1897-902.
16. Witayavanitkul N, Ait Mou Y, Kuster DW, Khairallah RJ, Sarkey J, Govindan S, et al. Myocardial infarction-induced N-terminal fragment of cardiac myosin-binding protein C (cMyBP-C) impairs myofilament function in human myocardium. *J Biol Chem.* 2014;289(13):8818-27.
17. Kobayashi T, Kosuge S, and Sugi H. Bidirectional functional communication between myosin subfragments 1 and 2 in skeletal muscle fibers. *Adv Exp Med Biol.* 1998;453:435-40.
18. Anderson RL, Trivedi DV, Sarkar SS, Henze M, Ma W, Gong H, et al. Deciphering the super relaxed state of human beta-cardiac myosin and the mode of action of mavacamten from myosin molecules to muscle fibers. *Proc Natl Acad Sci U S A.* 2018;115(35):E8143-E52.
19. Griffiths PJ, Isackson H, Pelc R, Redwood CS, Funari SS, Watkins H, et al. Synchronous in situ ATPase activity, mechanics, and Ca2+ sensitivity of human and porcine myocardium. *Biophys J.* 2009;97(9):2503-12.
20. Walklate J, Ferrantini C, Johnson CA, Tesi C, Poggesi C, and Geeves MA. Alpha and beta myosin isoforms and human atrial and ventricular contraction. *Cell Mol Life Sci.* 2021;78(23):7309-37.
21. Piroddi N, Belus A, Scellini B, Tesi C, Giunti G, Cerbai E, et al. Tension generation and relaxation in single myofibrils from human atrial and ventricular myocardium. *Pflugers Arch.* 2007;454(1):63-73.
22. Ma W, Lee KH, Delligatti CE, Davis MT, Zheng Y, Gong H, et al. The structural and functional integrities of porcine myocardium are mostly preserved by cryopreservation. *J Gen Physiol.* 2023;155(9).
23. Jiratrakanvong J, Shao J, Li J, Menendez Alvarez M, Li X, Das P, et al. MuscleX: data analysis software for fiber diffraction patterns from muscle. *Journal of synchrotron radiation.* 2024;31(Pt 5):1401-8.
24. Ma W, Henze M, Anderson RL, Gong H, Wong FL, Del Rio CL, et al. The Super-Relaxed State and Length Dependent Activation in Porcine Myocardium. *Circ Res.* 2021;129(6):617-30.
25. Huxley HE, Stewart A, Sosa H, and Irving T. X-ray diffraction measurements of the extensibility of actin and myosin filaments in contracting muscle. *Biophysical journal.* 1994;67(6):2411-21.
26. Ma W, McMillen TS, Childers MC, Gong H, Regnier M, and Irving T. Structural OFF/ON transitions of myosin in relaxed porcine myocardium predict calcium-

activated force. *Proceedings of the National Academy of Sciences of the United States of America.* 2023;120(5):e2207615120.

27. Ng R, Sewanan LR, Stankey P, Li X, Qyang Y, and Campbell S. Shortening Velocity Causes Myosin Isoform Shift in Human Engineered Heart Tissues. *Circ Res.* 2021;128(2):281-3.
28. Ochala J, Feng M, Wang Q, Chaami C, Nollet E, Lewis CTA, et al. Heterogeneous Dysregulation of Myosin Super-Relaxation and Energetics in Hypertrophic Cardiomyopathy. *Circ Heart Fail.* 2025:e012614.
29. Schneidereit D, Nubler S, Prolss G, Reischl B, Schurmann S, Muller OJ, et al. Optical prediction of single muscle fiber force production using a combined biomechatronics and second harmonic generation imaging approach. *Light Sci Appl.* 2018;7:79.
30. Wei EQ, Beyer M, Brown KJ, Bansbach AJ, Gorham JM, McDonough B, et al. Deep-learning analysis of 3D microarchitectural remodeling in hypertrophic cardiomyopathy. *Science.* 2026;391(6782):eady6443.
31. Livak KJ, and Schmittgen TD. Analysis of relative gene expression data using real-time quantitative PCR and the 2(-Delta Delta C(T)) Method. *Methods.* 2001;25(4):402-8.
32. Emter CA, and Bowles DK. Store-operated Ca(2+) entry is not essential for PDGF-BB induced phenotype modulation in rat aortic smooth muscle. *Cell Calcium.* 2010;48(1):10-8.
33. Marshall KD, Muller BN, Krenz M, Hanft LM, McDonald KS, Dellsperger KC, et al. Heart failure with preserved ejection fraction: chronic low-intensity interval exercise training preserves myocardial O2 balance and diastolic function. *J Appl Physiol (1985).* 2013;114(1):131-47.
34. Hawryluk N, Schlachter, S., Evanchik, M, Del Rio, C., Russell, A., Koch, K., Duvall, M., Luzzio, M. 1,4-Dihydroquinazoline derivatives for the treatment of HCM and their preparation. *International Patent Application No WO/2024/073426 Edgewise Therapeutics, Inc.* Published April 4, 2024.

Supplemental Figures and Tables

Supplemental Figure 1.

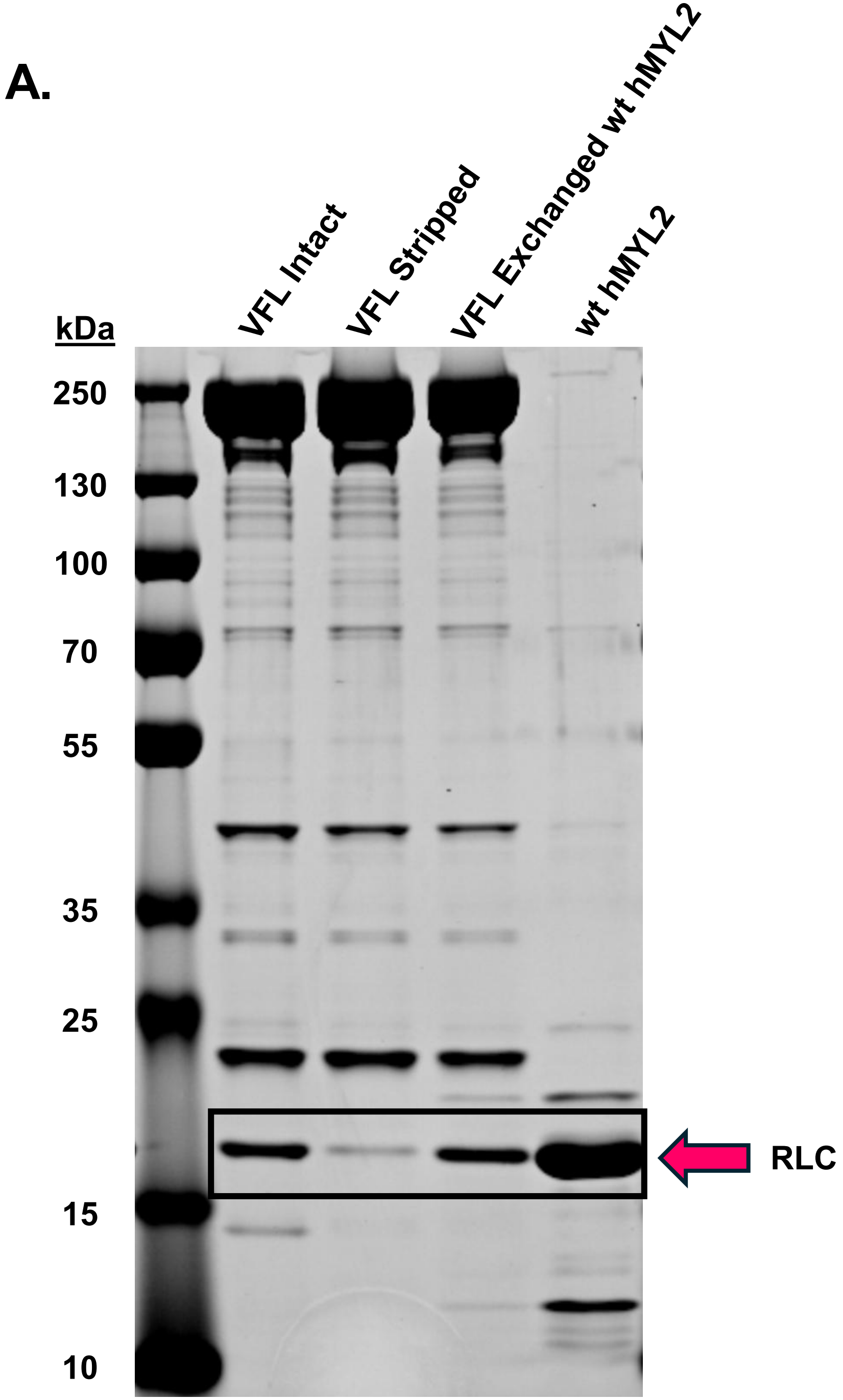

Supplemental Figure 2.

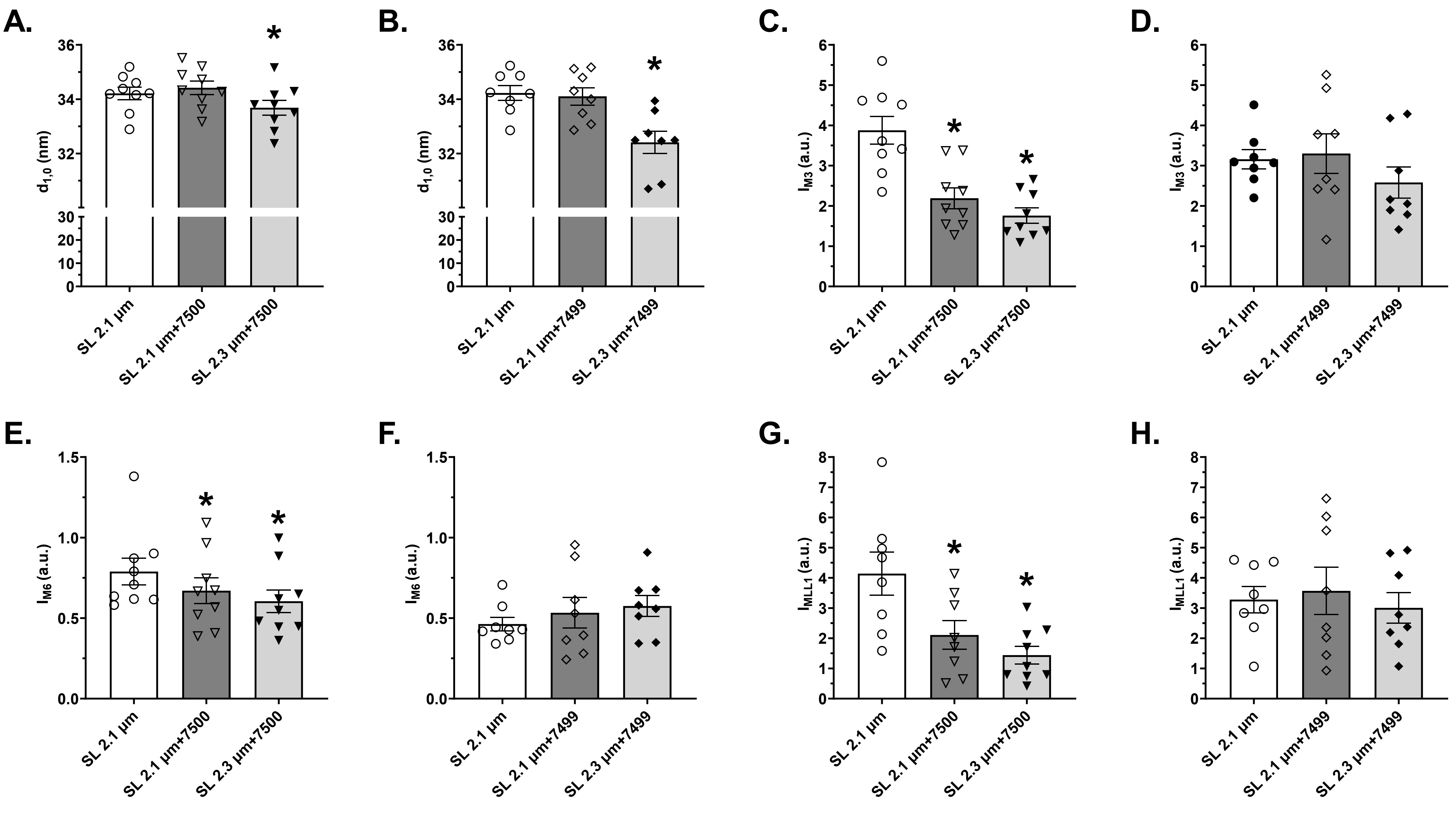

Supplemental Figure 3.

A.

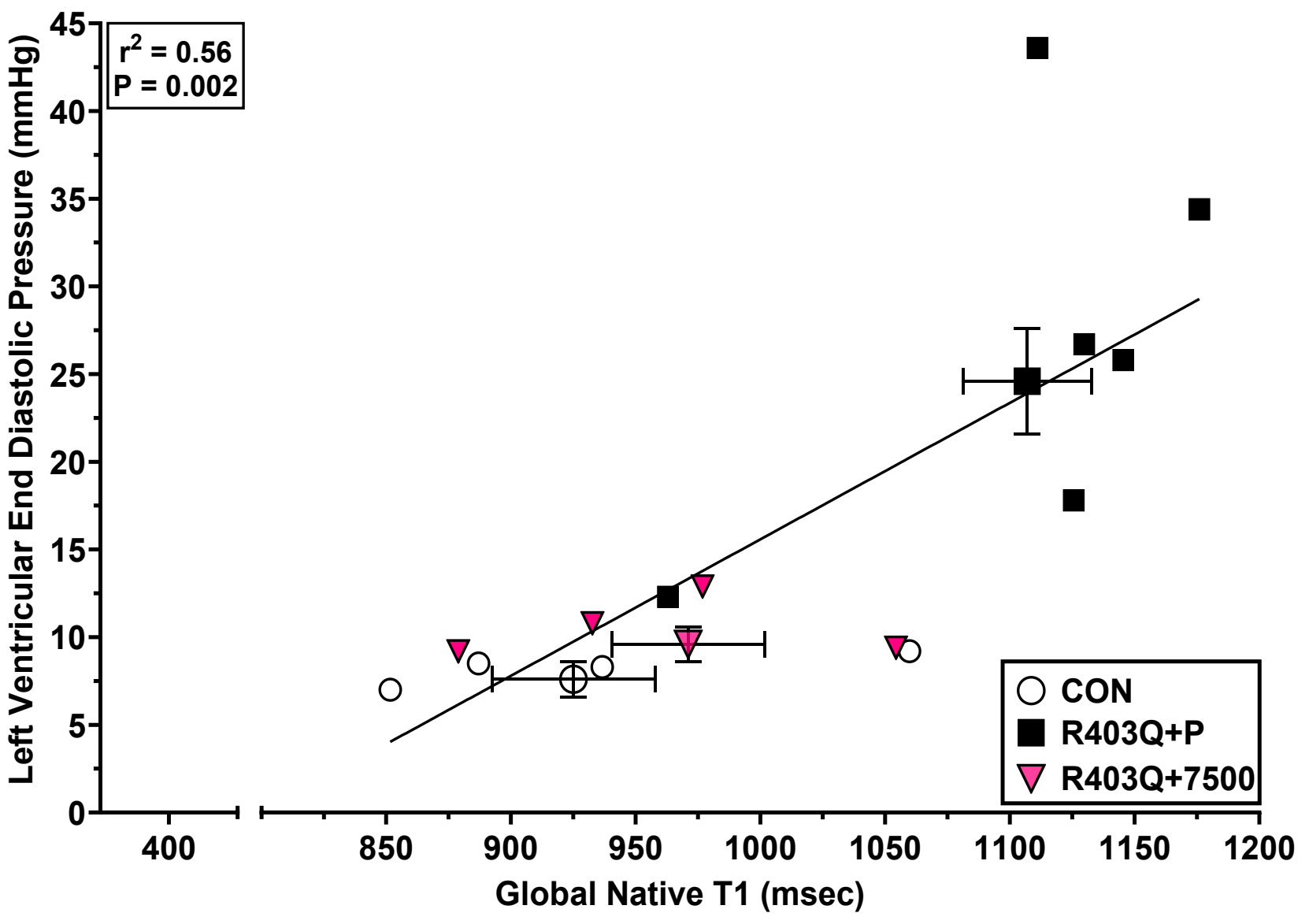

B.

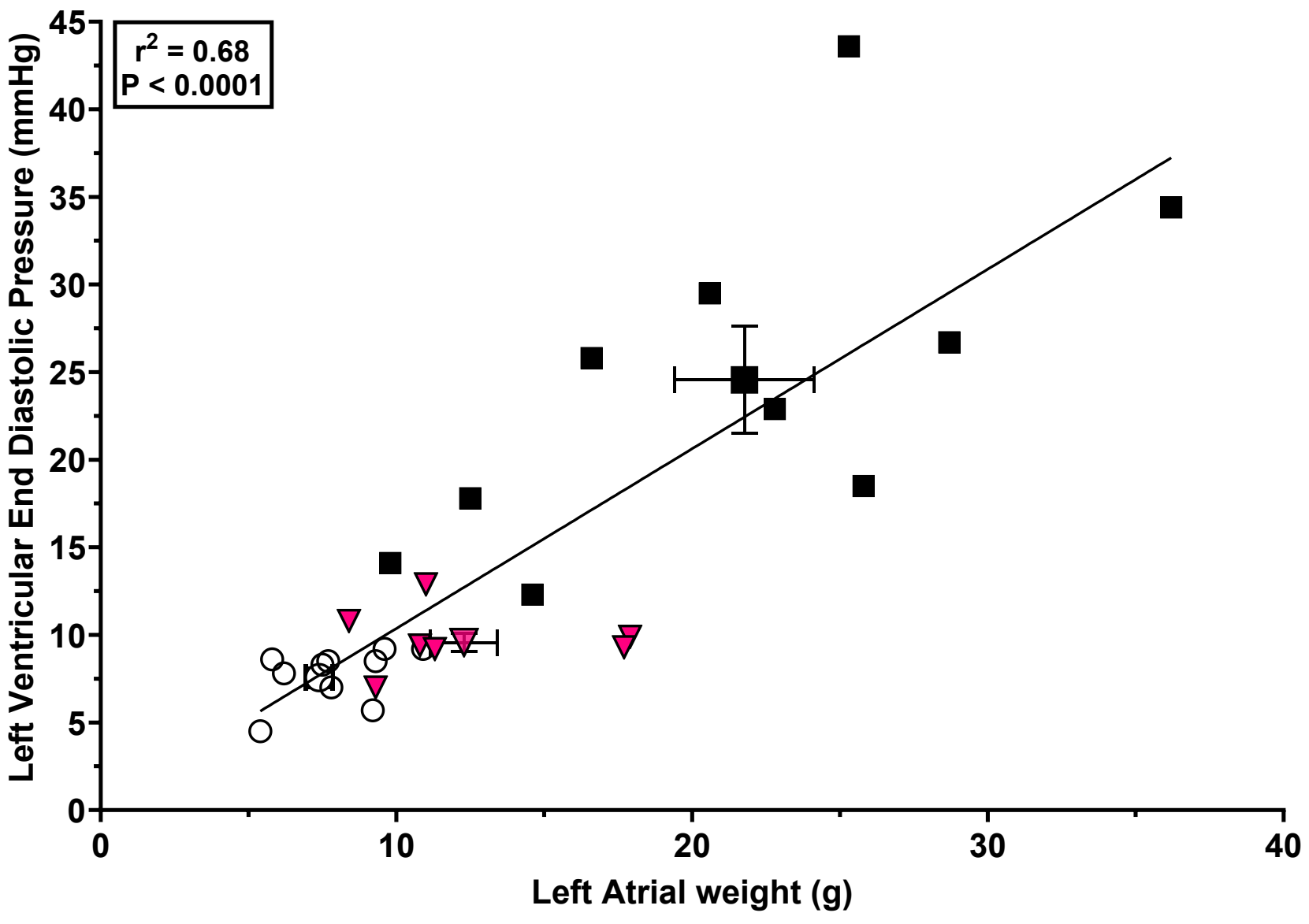

Supplemental Figure 4.

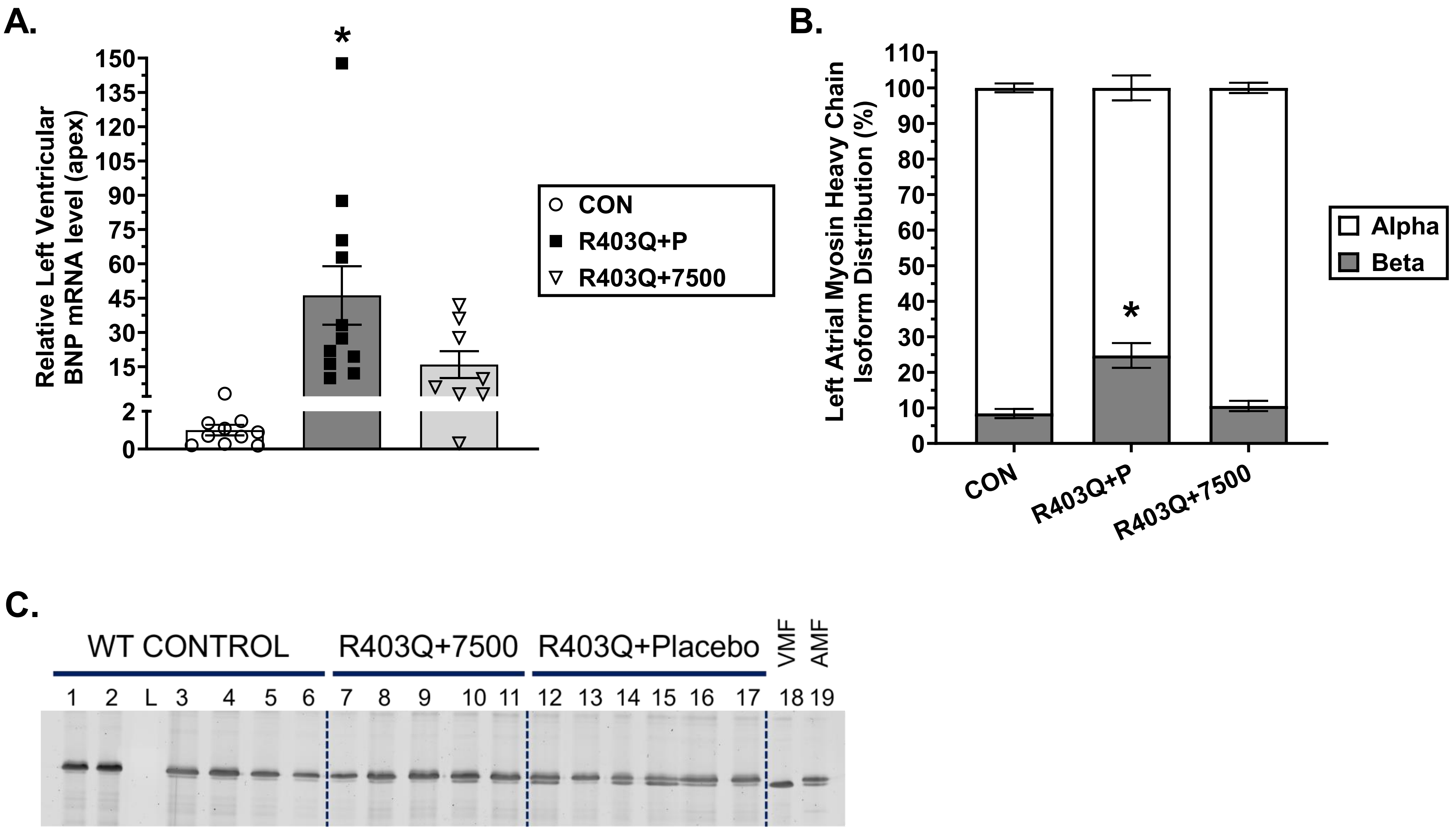

Supplemental Figure Legends

Suppl. Fig. 1 Representative Western blot highlighting the protein exchange of recombinant human RLC protein (encoded by human ventricle *MYL2*) on full length myosin.

Suppl. Fig. 2 *Stretch-dependent decreases in interfilament lattice spacing are independent of compound, while the intensity of M3, M6, and MLL1 meridional reflections show EDG-7500 disorders myosin heads along the myosin backbone in porcine LV myocardial strips.* **A-B,** EDG-7500 and EDG-7499 do not alter $d_{1,0}$ at sarcomere length 2.1 μm, and show similar decreases in $d_{1,0}$ when sarcomere length is stretched to 2.3 μm. **C-D,** EDG-7500 decreases I_{M3} at sarcomere length 2.1 μm and when stretched to 2.3 μm (**C**), whereas EDG-7499 has no effect (**D**). **E-F,** EDG-7500 decreases I_{M6} at sarcomere length 2.1 μm and when stretched to 2.3 μm (**E**), whereas EDG-7499 has no effect (**F**). **G-H,** EDG-7500 decreases I_{MLL1} at sarcomere length 2.1 μm and when stretched to 2.3 μm (**G**), whereas EDG-7499 has no effect (**H**). * P<0.05, repeated measures ANOVA vs. SL 2.1 μm.

Suppl. Fig. 3 *Chronic EDG-7500 normalizes hemodynamic-structural relationships in R403Q swine.* **A-B,** Diastolic hemodynamics are corelated to ventricular and atrial remodeling via both imaging and postmortem techniques. LV end diastolic pressure is positively correlated with both global native T1 time (**A**) and LA postmortem weight (**B**), with chronic EDG-7500 therapy shifting group means left and downward along this relationship towards normal CON levels in the R430Q+7500 group. P-values denote regression analysis.

Suppl. Fig. 4 *Chronic EDG-7500 therapy prevents increases in biomarkers associated with cardiovascular disease progression in R403Q swine.* **A**, Increased LV brain natriuretic peptide (BNP) mRNA level in the R403Q+P group is attenuated in R430Q+7500 animals. **B,** The HCM-mediated increase in LA β-myosin heavy chain isoform protein level in R403Q+P animals was also prevented in the R430Q+7500 group. **C,** Representative myosin heavy chain gel with samples from all groups presented in addition to control ventricular (VMF) and atrial (AMF) myofibril preps. *one way ANOVA vs. CON and R403Q+7500 (*P<0.05).

Supplemental Table 1.

Supplemental Table 1. Human clinical data from the patients used for the present study			
	SMP oHCM (MYBPC3/MYH7)	SMP oHCM (TNNT2/TNNI3)	SMN oHCM
N	12	8	7
Sex (male)	58%	50%	28%
Age (years)	50.2 ± 14.5	53.5 ± 18.6	61.4 ± 14.8
BMI ($kg.m^{-2}$)	26.4 ± 3.5	28.9 ± 5.5	27.3 ± 2.9
Diabetes (type 1 or 2)	0%	0%	0%
β-blockers	66%	12%	57%
Statins	8%	0%	0%
IVS (mm)	21.9 ± 4.8	20.7 ± 3.6	19.3 ± 3.1
LVEF (%)	65.6 ± 4.4	74.5 ± 23.3	66.6 ± 5.8
E/A ratio (a.u.)	1.12 ± 0.38	1.18 ± 0.61	1.27 ± 0.46
Diastolic dysfunction (grade)	1 (58%); 2 (42%)	1 (37%); 2 (38%); 3 (25%)	1 (14%); 2 (86%)
Rest LVOTg (mm Hg)	37.6 ± 29.6	70.7 ± 37.1	66.8 ± 47.2
Provoked LVOTg (mm Hg)	64.9 ± 22.2	76.3 ± 21.4	67.6 ± 32.1
