# Supplementary material for: Mechanistic diversity within RLC-dependent myosin ATPase inhibitors differentiates EDG-7500, a diastolic-selective cardiac sarcomere modulator
Source: JCI Insight. 2026 Sep 22;11(18):e203086. doi: 10.1172/jci.insight.203086 (PMC13596718; doi:10.1172/jci.insight.203086)

RLC exchange blot – area presented in Supplemental Figure 1

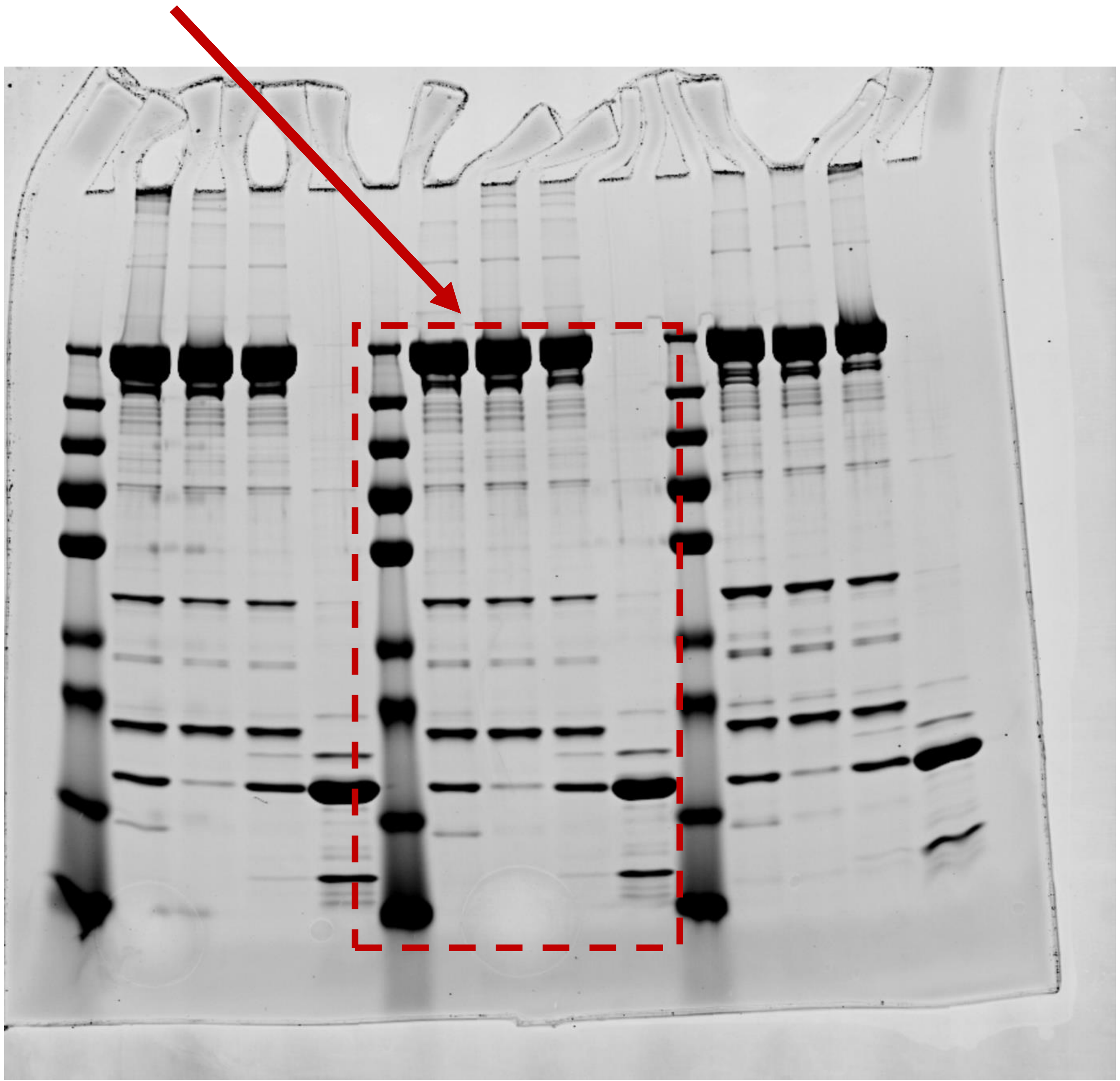

Left Atrial myosin heavy chain isoform – area presented in Supplemental Figure 4

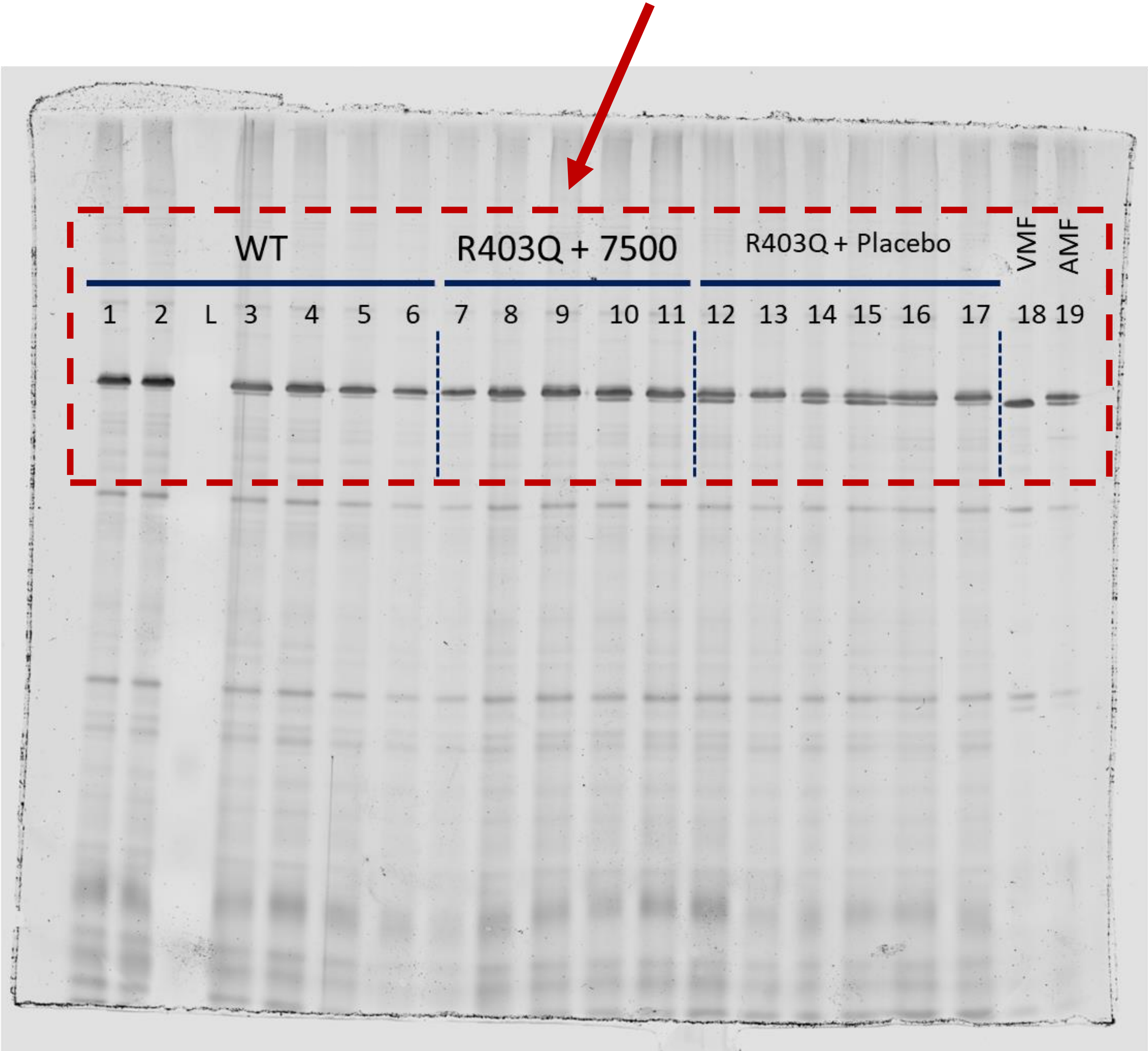

Supplement: Supplementary file 2 [file jciinsight-11-203086-s007.pdf]
